# Educational interventions targeting pregnant women to optimise the use of caesarean section: What are the essential elements? A qualitative comparative analysis

**DOI:** 10.1186/s12889-023-16718-0

**Published:** 2023-09-23

**Authors:** Rana Islamiah Zahroh, Katy Sutcliffe, Dylan Kneale, Martha Vazquez Corona, Ana Pilar Betrán, Newton Opiyo, Caroline S. E. Homer, Meghan A. Bohren

**Affiliations:** 1 Gender and Women’s Health Unit, Nossal Institute for Global Health, School of Population and Global Health, University of Melbourne, Melbourne, VIC Australia; 2 EPPI Centre, UCL Social Research Institute, University College London, London, UK; 3 Department of Sexual and Reproductive Health and Research, UNDP/UNFPA/UNICEF/WHO/World Bank Special Programme of Research, Development and Research Training in Human Reproduction (HRP), World Health Organization, Geneva, Switzerland; 4 Maternal, Child, and Adolescent Health Programme, Burnet Institute, Melbourne, VIC Australia

**Keywords:** Maternal health, Caesarean section, Qualitative comparative analysis, Complex intervention, Intervention implementation

## Abstract

**Background:**

Caesarean section (CS) rates are increasing globally, posing risks to women and babies. To reduce CS, educational interventions targeting pregnant women have been implemented globally, however, their effectiveness is varied. To optimise benefits of these interventions, it is important to understand which intervention components influence success. In this study, we aimed to identify essential intervention components that lead to successful implementation of interventions focusing on pregnant women to optimise CS use.

**Methods:**

We re-analysed existing systematic reviews that were used to develop and update WHO guidelines on non-clinical interventions to optimise CS. To identify if certain combinations of intervention components (e.g., how the intervention was delivered, and contextual characteristics) are associated with successful implementation, we conducted a Qualitative Comparative Analysis (QCA). We defined successful interventions as interventions that were able to reduce CS rates. We included 36 papers, comprising 17 CS intervention studies and an additional 19 sibling studies (e.g., secondary analyses, process evaluations) reporting on these interventions to identify intervention components. We conducted QCA in six stages: 1) Identifying conditions and calibrating the data; 2) Constructing truth tables, 3) Checking quality of truth tables; 4) Identifying parsimonious configurations through Boolean minimization; 5) Checking quality of the solution; 6) Interpretation of solutions. We used existing published qualitative evidence synthesis to develop potential theories driving intervention success.

**Results:**

We found successful interventions were those that leveraged social or peer support through group-based intervention delivery, provided communication materials to women, encouraged emotional support by partner or family participation, and gave women opportunities to interact with health providers. Unsuccessful interventions were characterised by the absence of at least two of these components.

**Conclusion:**

We identified four key essential intervention components which can lead to successful interventions targeting women to reduce CS. These four components are 1) group-based delivery, 2) provision of IEC materials, 3) partner or family member involvement, and 4) opportunity for women to interact with health providers. Maternal health services and hospitals aiming to better prepare women for vaginal birth and reduce CS can consider including the identified components to optimise health and well-being benefits for the woman and baby.

**Supplementary Information:**

The online version contains supplementary material available at 10.1186/s12889-023-16718-0.

## Introduction

In recent years, caesarean section (CS) rates have increased globally [1–4]. CS can be a life-saving procedure when vaginal birth is not possible; however, it comes with higher risks both in the short- and long-term for women and babies [1, 5]. Women with CS have increased risks of surgical complications, complications in future pregnancies, subfertility, bowel obstruction, and chronic pain [5–8]. Similarly, babies born through CS have increased risks of hypoglycaemia, respiratory problems, allergies and altered immunity [9–11]. At a population level, CS rates exceeding 15% are unlikely to reduce mortality rates [1, 12]. Despite these risks, an analysis across 154 countries reported a global average CS rate of 21.1% in 2018, projected to increase to 28.5% by 2030 [3].

There are many reasons for the increasing CS rates, and these vary between and within countries. Increasingly, non-clinical factors across different societal dimensions and stakeholders (e.g. women and communities, health providers, and health systems) are contributing to this increase [13–17]. Women may prefer CS over vaginal birth due to fear of labour or vaginal birth, previous negative experience of childbirth, perceived increased risks of vaginal birth, beliefs about an auspicious or convenient day of birth, or beliefs that caesarean section is safer, quick, and painless compared to vaginal birth [13–15].

Interventions targeting pregnant women to reduce CS have been implemented globally. A Cochrane intervention review synthesized evidence from non-clinical interventions targeting pregnant women and family, providers, and health systems to reduce unnecessary CS, and identified 15 interventions targeting women [18]. Interventions targeting women primarily focused on improving women’s knowledge around birth, improving women’s ability to cope during labour, and decreasing women’s stress related to labour through childbirth education, and decision aids for women with previous CS [18]. These types of interventions aim to reduce the concerns of pregnant women and their partners around childbirth, and prepare them for vaginal birth.

The effectiveness of interventions targeting women in reducing CS is mixed [18, 19]. Plausible explanations for this limited success include the multifactorial nature of the factors driving increases in CS, as well as the contextual characteristics of the interventions, which may include the study environment, participant characteristics, intensity of exposure to the intervention and method of implementation. Understanding which intervention components are essential influencers of the success of the interventions is conducive to optimising benefits. This study used a Qualitative Comparative Analysis (QCA) approach to re-analyse evidence from existing systematic reviews to identify essential intervention components that lead to the successful implementation of non-clinical interventions focusing on pregnant women to optimise the use of CS. Updating and re-analysing existing systematic reviews using new analytical frameworks may help to explore the heterogeneity in effects and ascertain why some studies appear to be effective while others are not.

## Methods

### Data sources, case selection, and defining outcomes

#### Developing a logic model

We developed a logic model to guide our understanding of different pathways and intervention components potentially leading to successful implementation (Additional file 1). The logic model was developed based on published qualitative evidence syntheses and systematic reviews [18, 20–24]. The logic model depicts the desired outcome of reduced CS rates in low-risk women (at the time of admission for birth, these women are typically represented by Robson groups 1–4 [25] and are women with term, cephalic, singleton pregnancies without a previous CS) and works backwards to understand what inputs and processes are needed to achieve the desired outcome. Our logic model shows multiple pathways to success and highlights the interactions between different levels of factors (women, providers, societal, health system) (Additional file 1). Based on the logic model, we have separated our QCA into two clusters of interventions: 1) interventions targeting women, and 2) interventions targeting health providers. The results of analysis on interventions targeting health providers have been published elsewhere [26]. The logic model was also used to inform the potential important components that influence success.

#### Identifying data sources and selecting cases

We re-analysed the systematic reviews which were used to inform the development and update of World Health Organization (WHO) guidelines. In 2018, WHO issued global guidance on non-clinical interventions to reduce unnecessary CS, with interventions designed to target three different levels or stakeholders: women, health providers, and health systems [27]. As part of the guideline recommendations, a series of systematic reviews about CS interventions were conducted: 1) a Cochrane intervention review of effectiveness by Chen et al. (2018) [18] and 2) three qualitative evidence syntheses exploring key stakeholder perspectives and experiences of interventions focusing on women and communities, health professionals, and health organisations, facilities and systems by Kingdon et al. (2018) [20–22]. Later on, Opiyo and colleagues (2020) published a scoping review of financial and regulatory interventions to optimise the use of CS [23].

Therefore, the primary data sources of this QCA are the intervention studies included in Chen et al. (2018) [18] and Opiyo et al. (2020) [23]. We used these two systematic reviews as not only they are comprehensive, but they were also used to inform the WHO guidelines development. A single intervention study is referred to as a “case”. Eligible cases were intervention studies focusing on pregnant women and aimed to reduce or optimise the use of CS. No restrictions on study design were imposed in the QCA. Therefore, we also assessed the eligibility of intervention studies excluded from Chen et al. (2018) [18] and Opiyo et al. (2020) [23] due to ineligible study designs (such as cohort study, uncontrolled before and after study, interrupted time series with fewer than three data points), as these studies could potentially show other pathways to successful implementation. We complemented these intervention studies with additional intervention studies published since the last review updates in 2018 and 2020, to include intervention studies that are likely to meet the review inclusion criteria for future review updates. No further search was conducted as QCA is suitable for medium-N cases, approximately around 10–50 cases, and inclusion of more studies may threaten study rigour [28].

Once eligible studies were selected, we searched for their ‘sibling studies’. Sibling studies are studies linked to the included intervention studies, such as formative research or process evaluations which may have been published separately. Sibling studies can provide valuable additional information about study context, intervention components, and implementation outcomes (e.g. acceptability, fidelity, adherence, dosage), which may not be well described in a single article about intervention effectiveness. We searched for sibling studies using the following steps: 1) reference list search of the intervention studies included in Chen et al. (2018) [18] and Opiyo et al. (2020) [23], 2) reference list search of the qualitative studies included in Kingdon et al. (2018) reviews [20–22]; and 3) forward reference search of the intervention studies (through “Cited by” function) in Scopus and Web of Science. Sibling studies were included if they included any information on intervention components or implementation outcomes, regardless of the methodology used. One author conducted the study screening independently (RIZ), and 10% of the screening was double-checked by a second author (MAB). Disagreements during screening were discussed until consensus, and with the rest of the author team if needed.

#### Defining outcomes

We assessed all outcomes related to the mode of birth in the studies included in the Chen et al. (2018) [18] and Opiyo et al. (2020) [23] reviews. Based on the consistency of outcome reporting, we selected “overall CS rate” as the primary outcome of interest due to its presence across studies. We planned to rank the rate ratio across these studies to select the 10 most successful and unsuccessful intervention studies. However, due to heterogeneity in how CS outcomes were reported across studies (e.g. odds ratios, rate ratios, percentages across different intervention stages), the final categorisation of successful or unsuccessful interventions is based on whether the CS rate decreased, based on the precision of the confidence interval or p-value (successful, coded as 1), or CS rate increased or did not change (unsuccessful, coded as 0).

#### Assessing risk of bias in intervention studies

All intervention studies eligible for inclusion were assessed for risk of bias. All studies included in Chen et al. (2018) and Opiyo et al. (2020) already had risk of bias assessed and reported [18, 23], and we used these assessments. Additional intervention studies outside the included studies on these reviews were assessed using the same tools depending on the type of evidence (two randomized controlled trials and one uncontrolled before and after study), and details of the risk of bias assessment results can be found in Additional file 2. We excluded studies with a high risk of bias to ensure that the analysis was based on high-quality studies and to enhance the ability of researchers to develop deep case knowledge by limiting the overall number of studies.

### Qualitative comparative analysis (QCA)

QCA was first developed and used in political sciences and has since been extended to systematic reviews of complex health interventions [24, 29–31]. Despite the term “qualitative”, QCA is not a typical qualitative analysis, and is often conceptualised as a methodology that bridges qualitative and quantitative methodologies based on its process, data used and theoretical standpoint [24]. Here, QCA is used to identify if certain configurations or combinations of intervention components (e.g. participants, types of interventions, contextual characteristics, and intervention delivery) are associated with the desired outcome [31]. These intervention components are referred to as “conditions” in the QCA methodology. Whilst statistical synthesis methods may be used to examine intervention heterogeneity in systematic reviews, such as meta-regression, QCA is a particularly suitable method to understand complex interventions like those aiming to optimise CS, as it allows for multiple overlapping pathways to causality [31]. Moreover, QCA allows the exploration of different combinations of conditions, rather than relying on a single condition leading to intervention effectiveness [31]. Although meta-regression allows for the assessment of multiple conditions, a sufficient number of studies may not be available to conduct the analysis. In complex interventions, such as interventions aiming to optimise the use of CS, single condition or standard meta-analysis may be less likely to yield usable and nuanced information about what intervention components are more or less likely to yield success [31].

QCA uses ‘set theory’ to systematically compare characteristics of the cases (e.g. intervention in the case of systematic reviews) in relation to the outcomes [31, 32]. This means QCA compares the characteristics of the successful ‘cases’ (e.g. interventions that are effective) to those unsuccessful ‘cases’ (e.g. interventions that are not effective). The comparison is conducted using a scoring system based on ‘set membership’ [31, 32]. In this scoring, conditions and outcomes are coded based on the extent to which a certain feature is present or absent to form set membership scores [31, 32]. There are two scoring systems in QCA: 1) crisp set QCA (csQCA) and 2) fuzzy set QCA (fsQCA). csQCA assigns binary scores of 0 (“fully out” to set membership for cases with certain conditions) and 1 (“fully in” to set membership for cases with certain conditions), while fsQCA assigns ordinal scoring of conditions and outcomes, permitting partial membership scores between 0 and 1 [31, 32]. For example, using fsQCA we may assign a five-level scoring system (0, 0.33, 0.5, 0.67, 1), where 0.33 would indicate “more out” than “in” to the set of membership, and 0.67 would indicate “more in” than “out”, and 0.5 would indicate ambiguity (i.e. a lack of information about whether a case was “in” or “out”) [31, 32]. In our analysis, we used the combination of both csQCA and fsQCA to calibrate our data. This approach was necessary because some conditions were better suited to binary options using csQCA, while others were more complex, depending on the distribution of cases, and required fsQCA to capture the necessary information. In our final analysis, however, the conditions run on the final analysis were all using the csQCA scoring system.

Two relationships can be investigated using QCA [24, 31]. First, if all instances of successful interventions share the same condition(s), this suggests these features are ‘necessary’ to trigger successful outcomes [24, 31]. Second, if all instances of a particular condition are associated with successful interventions, this suggests these conditions are ‘sufficient’ for triggering successful outcomes [24, 31]. In this QCA, we were interested to explore the relationship of sufficiency: that is, to assess the various combinations of intervention components that *can* trigger successful outcomes. We were interested in sufficiency because our logic model (explained further below) highlighted the multiple pathways that can lead to a CS and different interventions that may optimise the use of CS along those pathways, which suggested that it would be unlikely for all successful interventions to share the same conditions. We calculated the degree of sufficiency using consistency measures, which evaluate the frequency in which conditions are present when the desired outcome is achieved [31, 32]. The conditions with a consistency score of at least 0.8 were considered sufficient in triggering successful interventions [31, 32]. At present, there is no tool available for reporting guidelines in the re-analysis of systematic reviews using QCA, however, CARU-QCA is currently being developed for this purpose [33]. QCA was conducted using R programming software with a package developed by Thiem & Duşa (2013) and QCA with R guidebook [32]. QCA was conducted in six stages based on Thomas et al. (2014) [31] and explained below.

#### QCA stage 1: Identifying conditions, building data tables and calibration

We used a deductive and inductive process to determine the potential conditions (intervention components) that may trigger successful implementation. Conditions were first derived deductively using the developed logic model (Additional file 1). We then added additional conditions inductively using Intervention Component Analysis from the intervention studies [34], and qualitative evidence (“view”) synthesis [22] using Melendez-Torres’s (2018) approach [35]. Intervention Component Analysis is a methodological approach that examines factors affecting implementation through reflections from the trialist, which is typically presented in the discussion section of a published trial [34]. Examples of conditions identified in the Intervention Component Analysis include using an individualised approach, interaction with health providers, policies that encourage CS and acknowledgement of women’s previous birth experiences. After consolidating or merging similar conditions, a total of 52 conditions were selected and extracted from each included intervention and analysed in this QCA (Details of conditions and definitions generated for this study can be found in Additional files 3 and 4). We adapted the coding framework from Harris et al. (2019) [24] by adapting coding rules and six domains that were used, to organize the 52 conditions and make more sense of the data. These six domains are broadly classified as 1) context and participants, 2) intervention design, 3) program content, 4) method of engagement, 5) health system factors, and 6) process outcomes.

One author (RIZ) extracted data relevant to the conditions for each included study into a data table, which was then double-reviewed by two other authors (MVC, MAB). The data table is a matrix in which each case is represented in a row, and columns are used to represent the conditions. Following data extraction, calibration rules using either csQCA or fsQCA (e.g. group-based intervention delivery condition: yes = 1 (present), no = 0 (absent)) were developed through consultation with all authors. We developed a table listing the conditions and rules of coding the conditions, by either direct or transformational assignment of quantitative and qualitative data [24, 32] (Additional file 3 depicts the calibration rules). The data tables were then calibrated by applying scores, to explore the extent to which interventions have ‘set membership’ with the outcome or conditions of interest. During this iterative process, the calibration criteria were explicitly defined, emerging from the literature and the cases themselves. It is important to note, that maximum ambiguity is typically scored as 0.5 in QCA, however, we decided it would be more appropriate to assume that if a condition was not reported it was unlikely to be a feature of the intervention, so we treated not reported as “absence” that is we coded it 0.

#### QCA stage 2: Constructing truth tables

Truth tables are an analytical tool used in QCA to analyse associations between configurations of conditions and outcomes. Whereas the data table represents individual cases (rows) and individual conditions (columns) – the truth table synthesises this data to examine configurations – with each row representing a different configuration of the conditions. The columns indicate a) which conditions are featured in the configuration in that row, b) how many of the cases are represented by that configuration, and c) their association with the outcome.

We first constructed the truth tables based on context and participants, intervention designs, program content, and method of engagement; however, no configurations to trigger successful interventions were observed. Instead, we observed limited diversity, meaning there were many instances in which the configurations were unsupported by cases, likely due to the presence of too many conditions in the truth tables. We used the learning from these truth tables to return to the literature to explore potential explanatory theories about what conditions are important from the perspectives of participants and trialists to trigger successful interventions (adhering to the ‘utilisation of view’ perspective [35]). Through this process, we found that women and communities liked to learn new information about childbirth, and desired emotional support from partners and health providers while learning [22]. They also appreciated educational interventions that provide opportunities for discussion and dialogue with health providers and align with current clinical practice and advice from health providers [22]. Therefore, three models of truth tables were iteratively constructed and developed based on three important hypothesised theories about how the interventions should be delivered: 1) how birth information was provided to women, 2) emotional support was provided to women (including interactions between women and providers), and 3) a consolidated model examining the interactions of important conditions identified from model 1 and 2. We also conducted a sub-analysis of interventions targeting both women and health providers or systems (‘multi-target interventions’). This sub-analysis was conducted to explore if similar conditions were observed in triggering successful interventions in multi-target interventions, among the components for women only. Table 1 presents the list of truth tables that were iteratively constructed and refined.

**Table 1:** List of constructed truth tables constructed and refined

Model	Conditions considered
**Final models**	
Model 1: How birth information was provided to women	Information, communication, education (IEC) materials, antenatal education, psychoeducation, group-based intervention delivery
Model 2: Emotional support was provided to women	Partner or family member involvement, group-based intervention delivery, interaction with health providers
Consolidated model	IEC materials, interaction with health providers, partner or family members involvement, group-based intervention delivery
Sub-analysis of multi-target interventions	IEC materials, interaction with health providers, partner or family members involvement, group-based intervention delivery, multi-target intervention
**Other models explored but no configurations observed**	
Context and participants	Number of participants, partner or family members involvement, delivery at health facility, delivery at home or community, number of health facility, baseline CS rates
Intervention designs	Antenatal education, psychoeducation, decision aids, theory-driven, facilitators, group-based intervention delivery, personal based intervention delivery, IEC materials
Program content	Information about mode of birth, pain relief, mental health and coping strategies, partner’s roles, didactic-based intervention delivery, practice-based delivery
Methods of engagement	Recruitment through health facility, recruitment through advertisement, timing of engagement, frequency of engagement, duration of engagement, incentives, potential competing interests
Population of women targeted by the intervention	Partner or family member involvement, women with low risk pregnancies, women with previous CS, women with fear of birth, group-based intervention delivery, control group equivalent
Provision of emotional support for women (another pathway)	IEC materials can be taken home, partner or family member involvement, group-based intervention delivery

#### QCA stage 3: Checking quality of truth tables

We iteratively developed and improved the quality of truth tables by checking the configurations of successful and unsuccessful interventions, as recommended by Thomas et al. (2014) [31]. This includes by assessing the number of studies clustering to each configuration, and exploring the presence of any contradictory results between successful and unsuccessful interventions. We found contradictory configurations across the five truth tables, which were resolved by considering the theoretical perspectives and iteratively refining the truth tables.

#### QCA stage 4: Identifying parsimonious configurations through Boolean minimization

Once we determined that the truth tables were suitable for further analysis, we used Boolean minimisation to explore pathways resulting in successful intervention through the configurations of different conditions [31]. We simplified the “complex solution” of the pathways to a “parsimonious solution” and an “intermediate solution” by incorporating logical remainders (configurations where no cases were observed) [36].

#### QCA stage 5: Checking the quality of the solution

We presented the intermediate solution as the final solution instead of the most parsimonious solution, as it is most closely aligned with the underlying theory. We checked consistency and coverage scores to assess if the pathways identified were sufficient to trigger success. We also checked the intermediate solution by negating the outcome to see if it predicts the observed solutions.

#### QCA stage 6: Interpretation of solutions

We iteratively interpreted the results of the findings through discussions among the QCA team. This reflexive approach ensured that the results of the analysis considered the perspectives from the literature discourse, methodological approach, and that the results were coherent with the current understanding of the phenomenon.

## Results

### Overview of included studies

**Fig. 1:**
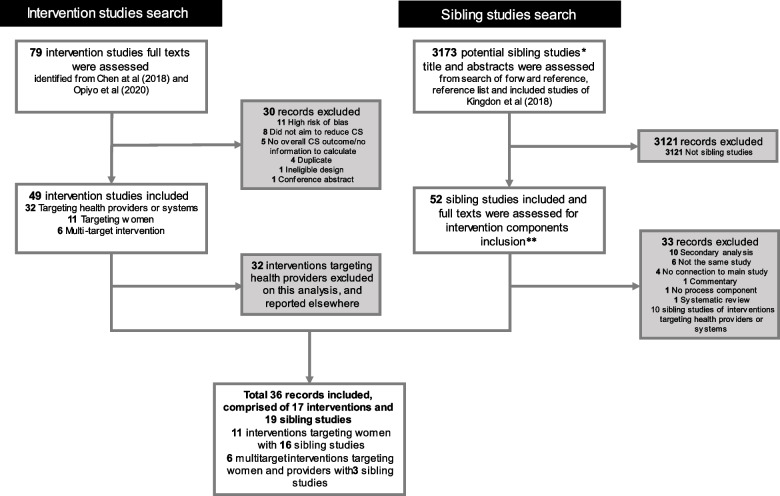
PRISMA flowchart. *Sibling studies: studies that were conducted in the same settings, participants, and timeframe; **Intervention components: information on intervention input, activities, and outputs, including intervention context and other characteristics

Out of 79 intervention studies assessed by Chen et al. (2018) [18] and Opiyo et al. (2020) [23], 17 intervention studies targeted women and are included, comprising 11 interventions targeting only women [37–43] and six interventions targeting both women and health providers or systems [44–49]. From 17 included studies, 19 sibling studies were identified [43, 49–67]. Thus, a total of 36 papers from 17 intervention studies are included in this QCA (See Fig. 1: PRISMA Flowchart).

The 11 interventions targeting women comprised of five successful interventions [37, 68–71] and six unsuccessful interventions [37–43] in reducing CS. Sixteen sibling studies were identified, from five out of 11 included interventions [37, 41, 43, 70, 71]. Included studies were conducted in six countries across North America (2 from Canada [38] and 1 from United States of America [71]), Asia–Pacific (1 from Australia [41]), 5 from Iran [39, 40, 68–70]), Europe (2 from Finland [37, 42], 1 from United Kingdom [43]). Six studies were conducted in high-income countries, while five studies were conducted in upper-middle-income countries (all from Iran). All 11 studies targeted women, with three studies also explicitly targeting women’s partners [68, 69, 71]. One study delivering psychoeducation allowed women to bring any family members to accompany them during the intervention but did not specifically target partners [37]. All 11 studies delivered childbirth education, with four delivering general antenatal education [38, 40, 68, 69], six delivering psychoeducation [37, 39, 41, 42, 70, 71], and one implementing decision aids [43]. All studies were included in Chen et al. (2018), and some risks of bias were identified [18] (Additional file 2).

The multi-target interventions consisted of five successful interventions [44–48] and one unsuccessful intervention [49]. Sibling studies were only identified from one study [48]. The interventions were delivered in five countries across: South America (1 from Brazil [46]), Asia–Pacific (4 from China [44, 45, 47, 49]), Europe (1 from Italy [48], 1 from Ireland [48], and 1 from Germany [48]). Three studies were conducted in high-income countries and five studies in upper middle-income countries. The multi-target interventions targeted women, health providers and health organisations. For this analysis, however, we only consider the components of the intervention that targeted women, which was typically childbirth education. One study came from Chen et al. (2018) [18] and was graded as having some concerns [47], two studies from Opiyo et al. (2020) [23] were graded as having no serious concerns [45, 46], and three studies are newly published studies assessed as low [44] and some concerns about risk of bias [48, 49] Table 2 and 3 show characteristics of included studies.

**Table 2:** Summary of characteristics of included intervention studies

Characteristic	N of studies (%), *N* = 17	Studies
Setting		
High-income countries	7 (41.2%)	[37, 38, 41–43, 71]
Upper middle-income countries	10 (58.8%)	[39, 40, 44–49, 60, 68, 69]
Data collection period		
< 2000	2 (11.8%)	[38, 42]
2000–2010	4 (23.5%)	[37, 43, 70, 71]
> 2010*	8 (47.0%)	[39, 41, 44–49]
Unclear/NI	3 (17.7%)	[40, 68, 69]
Study design		
Randomized controlled trial	13 (76.5%)	[37–43, 49, 60, 66, 68, 69, 71]
Before and after	3 (17.7%)	[44, 45, 47]
Interrupted time series	1 (5.9%)	[46]
Sample size		
< 100	2 (11.8%)	[40, 68]
100–1000	8 (47.0%)	[37, 39, 41–43, 69–71]
> 1000	7 (41.2%)	[38, 44–49]
Type of women		
Women with low-risk pregnancy	9 (52.9%)	[39, 44–47, 49, 68, 69, 71]
Women with fear of childbirth	5 (29.4%)	[37, 40–42, 70]
Women with previous CS	3 (17.7%)	[38, 43, 48]
Baseline CS rate		
< 30%	2 (11.8%)	[37, 43]
30–40%	0 (0%)	-
> 40%	10 (58.8%)	[39, 40, 44–47, 49, 66, 68, 69]
Unclear/NI	5 (29.4%)	[38, 41, 42, 70, 71]
Outcomes		
Successful interventions	10 (58.8%)	[37, 44–48, 68–71]
Unsuccessful interventions	7 (41.2%)	[38–43, 49]

^*^Study started before 2010 and ended after 2010 is categorised as > *2010*[45, 47]

**Table 3:** Characteristics of included intervention studies

Author	Sibling studies	Country & study years	Intervention	Comparison	Methods	Settings	Type of women	Study sample	Baseline CS rates	Relative effect	CS outcomes	Risk of Bias*
Interventions targeting women												
Fraser 1997 [38]	None	Canada 1992—1994	Individualised prenatal education and support programme versus written information in pamphlet	Pamphlet	Randomized controlled trial	Hospital	Women with previous CS	1,301	Baseline CS rates were not stated but high preference in region of CS was cited	RR 0.88 (0.58–1.33)	Little or no difference	Some concerns
Fenwick 2015 [41]	4 studies: protocol [50], randomized controlled trial [51], cost-effectiveness [52], economic evaluation [53]	Australia 2012—2013	Midwife psychoeducation by telephone	Usual maternity care	Randomized controlled trial	Delivered by telephone	Women with fear of childbirth	339	Baseline CS rates were not stated but increasing CS in the country was cited	RR 0.81 (0.56–1.18)	Little or no difference	Some concerns
Masoumi 2016 [39]	None	Iran 2012—2013	Antenatal education programme for physiologic childbirth	Routine prenatal education	Randomized controlled trial	Hospital	Women with low-risk pregnancy	150	48% at country level	RR 1.03 (0.72–1.49)	Little or no difference	Some concerns
Montgomery 2007 [43]	7 studies: protocol [43], formative qualitative [54, 55], cost analysis [56], qualitative evaluation [57, 58], observational [59]	UK 2004—2006	Computer decision aids versus usual care	Usual care	Randomized controlled trial	Maternity units	Women with previous CS	742	22%-25% at facility level	Decision analysis group: RR 0.90 (0.79–1.02) & information group: RR 1.02 (0.90–1.14)	Little or no difference	Some concerns
Navaee 2015 [40]	None	Iran Unclear	Role-play education versus standard education using lectures	Lecture education group	Blind clinical trial	Health centres	Women with fear of childbirth	67	47% at country level	RR 0.66 (0.39–1.12)	Little or no difference	Some concerns
Saisto 2001 [42]	None	Finland 1996—1999	Intensive group therapy (cognitive behavioural therapy and childbirth psychotherapy)	Conventional therapy	Randomized controlled trial	Outpatient clinic	Women with fear of childbirth	176	Baseline CS rates were not stated but high preference for CS was cited among women with fear of birth (region not stated)	RR 0.90 (0.65–1.36)	Little or no difference	Some concerns
Bastani 2005 [70]	1 study: randomized controlled trial [60]	Iran 2002—2003	Nurse-led applied relaxation (breathing techniques & muscle relaxation) training programme	Routine prenatal care	Randomized controlled trial	Prenatal clinics	Women with fear of childbirth	110	Baseline CS rates were not stated but increasing CS worldwide was cited	RR 0.22 (0.11–0.43)	Decrease	Some concerns
Feinberg 2015 [71]	2 studies: randomized controlled trials [61, 62]	USA 2004—2006	Psychosocial couple-based prevention programme	Routine care (no educational classes)	Randomized controlled trial	Unclear	Women with low-risk pregnancy	169	Not stated	RR 0.53 (0.32–0.90)	Decrease	Some concerns
Rouhe 2013 [37]	2 studies: randomized controlled trials [63, 64]	Finland 2007—2009	Psychoeducation	Conventional care group	Randomized controlled trial	Hospital	Women with fear of childbirth	271	19.9% at country level	RR 0.70 (0.49–1.01)	Decrease	Some concerns
Sharifirad 2013 [68]	None	Iran Unclear	Prenatal education for husbands	Unclear	Randomized controlled trial	Unclear	Women with low-risk pregnancy	88	41.6% at province level	CS rate in case and control groups was 29.5% and 50% (P < 0.05)	Decrease	Some concerns
Valiani 2014 [69]	None	Iran Unclear	Childbirth training workshop	Mothers (alone), couples (mothers and partners), and control	Randomized controlled trial	Healthcare centres	Women with low-risk pregnancy	180	60% at province level	Mother group: RR 0.55 (0.33–0.89) & couple group: RR 0.59 (0.37–0.94)	Decrease	Some concerns
Interventions targeting women and health providers												
Zhang 2020 [49]	None	China 2015—2017	Targeted health education to pregnant women, improved hospital CS policy, and training of midwives/doulas for 8 months	Usual practice	Randomized controlled trial	Tertiary and secondary hospitals	Women with low-risk pregnancy	10,752	42.50% at facility level	OR = 0.92; 95% CI 0.73, 1.15	Little or no difference	Some concerns
Borem 2020 [46]	None	Brazil 2014—2016	A coalition of stakeholders, empowerment of pregnant women to choose mode of delivery, psychologic birth promotion, information system for providers	Baseline	Interrupted time series	Hospitals	Women with low-risk pregnancy	119,378	78.3% at facility level	Vaginal deliveries RR 1.62 (95% CI 1.27 to 2.07, p < 0.001)	Decrease	Not serious
Clarke 2020 [48]	3 studies: process evaluation [65], protocol [66], formative qualitative [67]	Italy, Ireland, Germany 2012—2016	Education of clinicians and women with one previous CS, appointment of opinion leaders, audit/peer review, and joint discussions by women and clinicians	Usual practice	Randomized controlled trial	Maternity units	Women with previous CS	2,002	VBAC rates < 35% at facility level	RR 0.9 (95%CI 0.85 to 0.98)	Decrease	Some concerns
Runmei 2012	None	China 2005—2011	Continuous quality improvement programme (education for staff and women, audits, public health education, monitoring CS rates, and neonatal outcomes)	Baseline	Controlled before-after study	Regional referral centre	Women with low-risk pregnancy	25,280	53.5%-56.1% at facility level	OR 0.56 (0.52–0.59)	Decrease	Some concerns
Yu 2017 [45]	None	China 2006—2014	Face-to-face weekly educational meetings between patients and hospital staff, training for providers, new regulations adoption and projects on CS (i.e. encourage mothers to choose vaginal delivery, strictly control indications for CS and maternal request for CS)	Baseline	Pre-post intervention study	Tertiary public hospitals	Women with low-risk pregnancy	131,312	55–56% at facility level	Overall CS rate declined by 1.29% (*p* = 0.002)	Decrease	Not serious
Xia 2019 [44]	None	China 2010—2016	Programs for population health education, skills training for healthcare professionals, equipment and technical support for local healthcare facilities, and capacity building for the maternal near-miss care system	Baseline	Uncontrolled before-after study	Hospitals and community	Women with low-risk pregnancy	1,923,687	42% at facility level	Decreasing trend in the monthly CS rates (Z = 75.067, p < 0.001)	Decrease	Low risks of bias

^*^See Additional file 2 for details of risk bias assessment

The childbirth education interventions included information about mode of birth, birth process, mental health and coping strategies, pain relief methods, and partners’ roles in birth. Most interventions were delivered in group settings, and only in three studies they were delivered on a one-to-one basis [38, 41, 42]. Only one study explicitly stated that the intervention was individualised to a woman’s unique needs and experiences [38].

Overall, there was limited theory used to design interventions among the included studies: less than half of interventions (7/17) explicitly used theory in designing the intervention. Among the seven interventions that used theory in intervention development, the theories included the health promotion-disease prevention framework [38], midwifery counselling framework [41], cognitive behavioural therapy [42], Ost’s applied relaxation [70], conceptual model of parenting [71], attachment and social cognitive theories [37], and healthcare improvement scale-up framework [46]. The remaining 10 studies only relied on previously published studies to design the interventions. We identified very limited process evaluation or implementation outcome evidence related to the included interventions, which is a limitation of the field of CS and clinical interventions more broadly.

### Qualitative comparative analysis

#### Model 1 – How birth information was provided to women

Model 1 is constructed based on the finding from Kingdon et al. (2018) [22] that women and communities enjoy learning new birth information, as it opens up new ways of thinking about vaginal birth and CS. Learning new information allows them to understand better the benefits and risks of CS and vaginal births, as well as increase their knowledge about CS [22].

We used four conditions in constructing model 1 truth table: 1) the provision of information, education, and communication (IEC) materials on what to expect during labour and birth, 2) type of education delivered (antenatal education or psychoeducation), and 3) group-based intervention delivery. We explored this model considering other conditions, such as type of information provided (e.g. information about mode of birth including birth process, mental health and coping strategies, pain relief), delivery technique (e.g. didactic, practical) and frequency and duration of intervention delivery; however these additional conditions did not result in configurations.

Of 16 possible configurations, we identified seven configurations (Table 4). The first two row shows perfect consistency of configurations (inclusion = 1) in five studies [37, 68–71] in which all conditions are present, except antenatal education or psychoeducation. The remaining configurations are unsuccessful interventions. Interestingly, when either IEC materials or group-based intervention delivery are present (but not both), implementation is likely to be unsuccessful (rows 3–7).

**Table 4:** Truth table model 1 – how birth information was provided to women

Row	Communication (IEC) materials	Antenatal Education	Psychoeducation	Group based intervention delivery	Outcome	Number of cases	InclS*	PRI**	Cases
1	1	0	1	1	1	3	1	1	Bastani (2005), [70] Feinberg (2015), [71] Rouhe (2013) [37]
2	1	1	0	1	1	2	1	1	Sharifirad (2013), [68] Valiani (2014) [69]
3	0	0	1	1	0	1	0	0	Masoumi (2016) [39]
4	0	1	0	1	0	1	0	0	Navaee (2015) [40]
5	1	0	0	0	0	1	0	0	Montgomery (2007) [43]
6	1	0	1	0	0	2	0	0	Fenwick (2015), [41] Saisto (2001) [42]
7	1	1	0	0	0	1	0	0	Fraser (1997)

^*^Inclusion score (InclS), also known as consistency, indicates the degree to which the evidence is consistent with the hypothesis that there is sufficient relation between the configuration and the outcome; **Proportional Reduction in Inconsistency (PRI) refers to the extent in which a configuration is sufficient in triggering successful outcome as well as the negation of the outcome

**Fig. 2:**
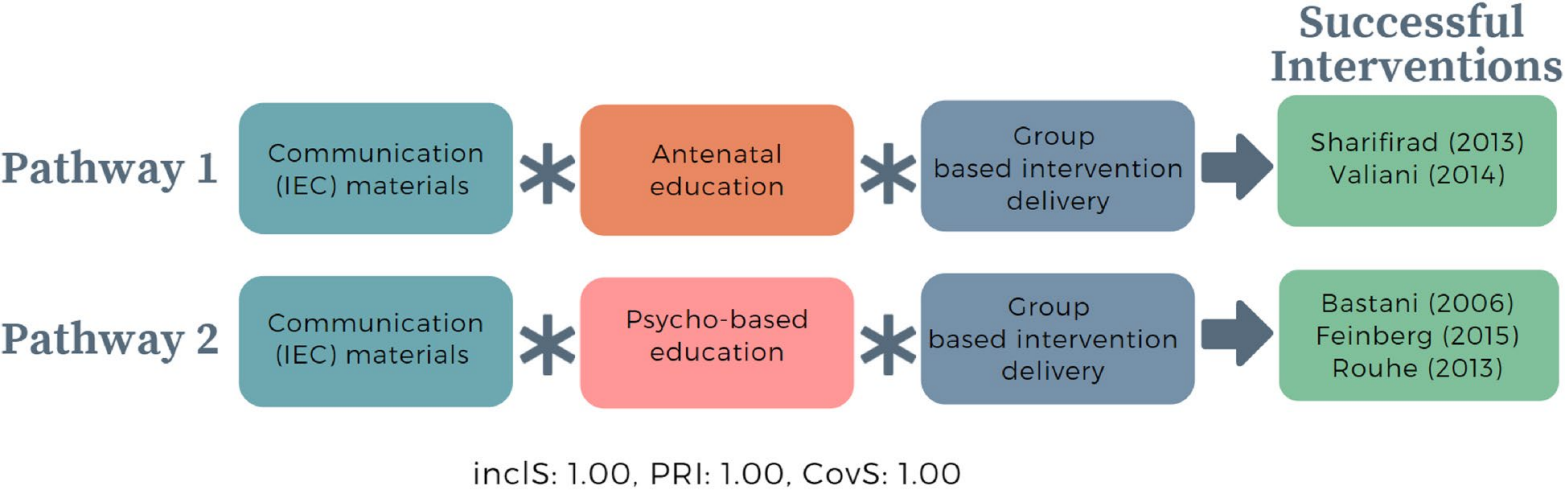
Intermediate pathways from model 1 that trigger successful interventions targeting pregnant women to optimise CS. In QCA, asterisk (*) denotes an ‘AND’ relationship; Inclusion score (InclS), also known as consistency, indicates the degree to which the evidence is consistent with the hypothesis that there is sufficient relation between the configuration and the outcome; Proportional Reduction in Inconsistency (PRI) refers to the extent in which a configuration is sufficient in triggering successful outcome as well as the negation of the outcome; Coverage score (CovS) refers to percentage of cases in which the configuration is valid

Boolean minimisation identified two intermediate pathways to successful interventions (Fig. 2). The two pathways are similar, except for one condition: type of education. The antenatal education or psychoeducation materials is the content tailored to the type of women they target. Therefore, from the two pathways, we can see that the presence of distribution of IEC materials on birth information and group-based intervention delivery of either antenatal education to the general population of women (e.g. not groups of women with specific risks or conditions) or psychoeducation to women with fear of birth trigger successful interventions. From this solution, we can see that the successful interventions are consistently characterised by the presence of both IEC materials and group-based intervention delivery.

#### Model 2 – Emotional support was provided to women

Model 2 was constructed based on the theory that women desire emotional support alongside the communication of information about childbirth [22]. This includes emotional support from husbands or partners, health professional, or doulas [22]. Furthermore, Kingdon et al. (2018) describe the importance of two-way conversation and dialogue between women and providers during pregnancy care, particularly to ensure the opportunity for discussion [22]. Interventions may generate more questions than they answered, creating the need and desire of women to have more dialogue with health professionals [22]. Women considered intervention content to be most useful when it complements clinical care, is consistent with advice from health professionals and provides a basis for more informed, meaningful dialogue between women and care providers [22].

Based on this underlying theory, we constructed model 3 truth table by considering three conditions representative of providing emotional support to women, including partner or family member involvement, group-based intervention delivery which provide social or peer support to women, and opportunity for women to interact with health providers. Of 8 possible configurations, we identified six configurations (Table 5). The first three rows represent successful interventions with perfect consistency (inclusion = 1). The first row shows successful interventions with all conditions present. The second and third row shows successful interventions with all conditions except partner or family member involvement or interaction with health providers. The remaining rows represent unsuccessful interventions, where at least two conditions are absent.

**Table 5:** Truth table model 2 – Emotional support was provided to women

Row	Group-based intervention delivery	Partner or family member involvement	Interaction with health provider	Outcome	Number of cases	inclS*	PRI**	Cases
1	1	1	1	1	2	1	1	Valiani (2014), [69] Rouhe (2013) [37]
2	1	0	1	1	1	1	1	Bastani (2005) [70]
3	1	1	0	1	2	1	1	Sharifirad (2013), [68] Feinberg (2015) [71]
4	0	0	0	0	1	0	0	Montgomery (2007) [43]
5	0	0	1	0	3	0	0	Fraser (1997), Fenwick (2015) [41]
6	1	0	0	0	2	0	0	Masoumi (2016), [39] Navaee (2015) [40]

^*^Inclusion score (InclS), also known as consistency, indicates the degree to which the evidence is consistent with the hypothesis that there is sufficient relation between the configuration and the outcome; **Proportional Reduction in Inconsistency (PRI) refers to the extent in which a configuration is sufficient in triggering successful outcome as well as the negation of the outcome

**Fig. 3:**
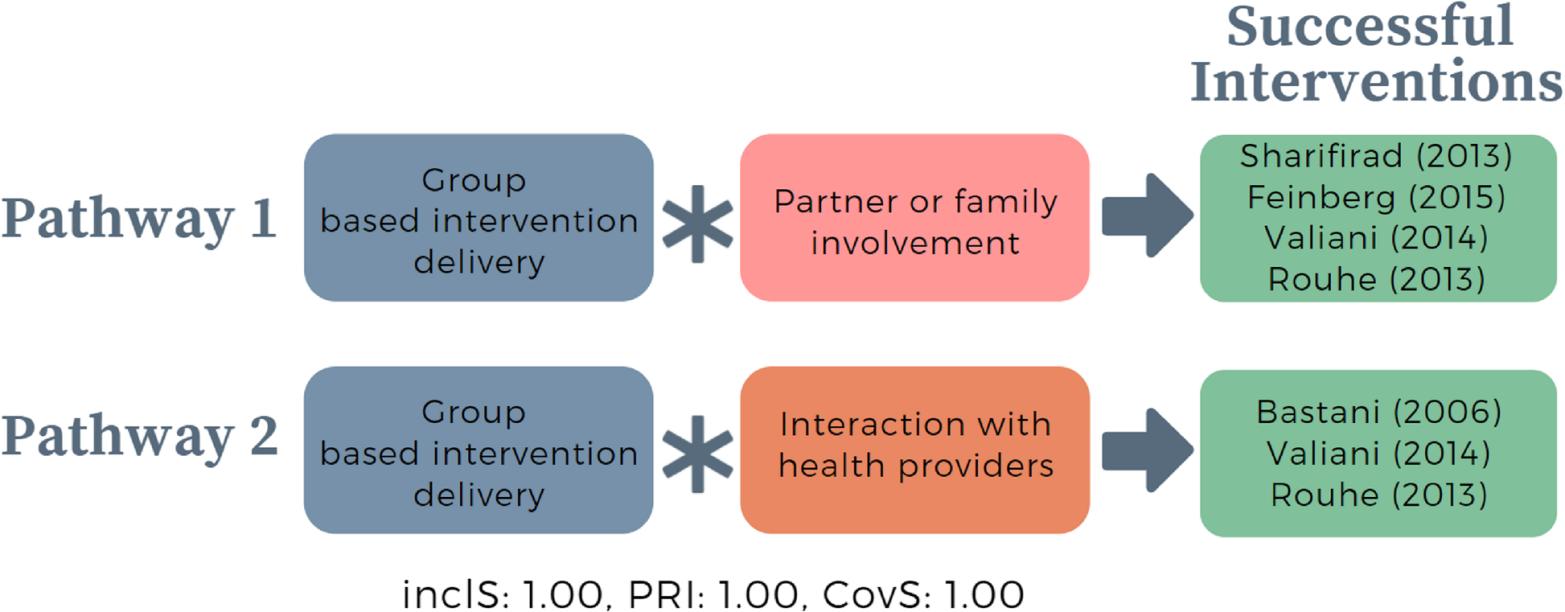
Intermediate pathways from model 2 that trigger successful interventions targeting pregnant women to optimise CS. In QCA, asterisk (*) denotes an ‘AND’ relationship; Inclusion score (InclS), also known as consistency, indicates the degree to which the evidence is consistent with the hypothesis that there is sufficient relation between the configuration and the outcome; Proportional Reduction in Inconsistency (PRI) refers to the extent in which a configuration is sufficient in triggering successful outcome as well as the negation of the outcome; Coverage score (CovS) refers to percentage of cases in which the configuration is valid

Boolean minimisation identified two intermediate pathways to successful interventions (Fig. 3). In the first pathway, the partner or family members involvement and group-based intervention delivery enable successful interventions. In the second pathway, however, when partner or family members are not involved, successful interventions can happen only when interaction with health providers is included alongside group-based intervention. From these two pathways, we can see that group-based intervention, involvement of partner and family member, and opportunity for women to interact with providers seem to be important in driving intervention success.

#### Consolidated model – Essential conditions to prompt successful interventions focusing on women

Using the identified important conditions observed in models 1 and 2, we constructed a consolidated model to examine the final essential conditions which could prompt successful educational interventions targeting women. We merged and tested four conditions: the provision of IEC materials on what to expect during labour and birth, group-based intervention delivery, partner or family member involvement, and opportunity for interaction between women and health providers.

Of the 16 possible configurations, we identified six configurations (Table 6). The first three rows show configurations resulting in successful interventions with perfect consistency (inclusion = 1). The first row shows successful interventions with all conditions present; the second and third rows show successful interventions with all conditions present except interaction with health providers or partner or family member involvement. The remaining three rows are configurations of unsuccessful interventions, missing at least two conditions, including the consistent absence of partner or family member involvement.

**Table 6:** Truth table of consolidated model – essential conditions to prompt successful educational interventions focusing on pregnant women

Row	Group based delivery	Communication (IEC) materials	Partner of family member involvement	Interaction with providers	Outcome	Number of cases	inclS*	PRI**	Cases
1	1	1	1	1	1	2	1	1	Valiani (2014),[69] Rouhe (2013) [37]
2	1	1	1	0	1	2	1	1	Sharifirad (2013), [68] Feinberg (2015) [71]
3	1	1	0	1	1	1	1	1	Bastani (2005) [70]
4	0	1	0	0	0	1	0	0	Montgomery (2007) [43]
5	0	1	0	1	0	3	0	0	Fraser (1997), Fenwick (2015), [41] Saisto (2001) [42]
6	1	0	0	0	0	2	0	0	Masoumi (2016), [39] Navaee (2015) [40]

^*^Inclusion score (InclS), also known as consistency, indicates the degree to which the evidence is consistent with the hypothesis that there is sufficient relation between the configuration and the outcome; **Proportional Reduction in Inconsistency (PRI) refers to the extent in which a configuration is sufficient in triggering successful outcome as well as the negation of the outcome

**Fig. 4:**
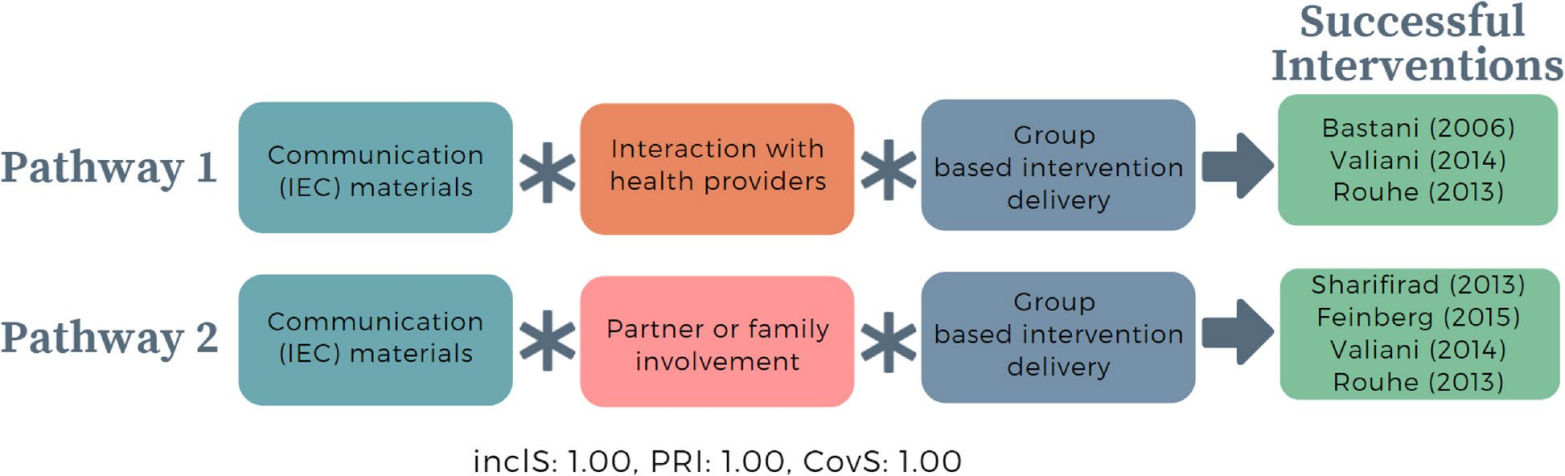
Intermediate pathways from consolidated model that trigger successful interventions targeting pregnant women to optimise CS. In QCA, asterisk (*) denotes an ‘AND’ relationship; Inclusion score (InclS), also known as consistency, indicates the degree to which the evidence is consistent with the hypothesis that there is sufficient relation between the configuration and the outcome; Proportional Reduction in Inconsistency (PRI) refers to the extent in which a configuration is sufficient in triggering successful outcome as well as the negation of the outcome; Coverage score (CovS) refers to percentage of cases in which the configuration is valid.

Boolean minimisation identified two intermediate pathways to successful intervention (Fig. 4). The first pathway shows that the opportunity for women to interact with health providers, provision of IEC materials, and group-based intervention delivery prompts successful interventions. The second pathway, however, shows that when there is no opportunity for women to interact with health providers, it is important to have partner or family member involvement alongside group-based intervention delivery and provision of IEC materials. These two pathways suggest that the delivery of educational interventions accompanied by provision of IEC materials and presence of emotional support for women during the intervention is important to trigger successful interventions. These pathways also emphasise that emotional support for women during the intervention can come from either partner, family member, or health provider. For the consolidated model, we did not simplify the solution further, as the intermediate solution is more theoretically sound compared to the most parsimonious solution.

#### Sub-analysis – Interventions targeting both women and health providers or systems

In this sub-analysis, we run the important conditions identified from the consolidated model, added condition of multi-target intervention, and applied it to 17 interventions: 11 interventions targeting women, and six interventions targeting both women and health providers or systems (multi-target interventions).

Of 32 possible configurations, we identified eight configurations (Table 7). The first four rows show configurations with successful interventions with perfect consistency (inclusion = 1). The first row is where all the multi-target interventions are clustered, except the unsuccessful intervention Zhang (2020) [49], and where all the conditions are present. All the conditions in the second to fourth rows are present, except multi-target interventions (all rows), interaction with health providers (third row) and partner and family member involvement (fourth row). The remaining rows are all configurations to unsuccessful interventions, where at least three conditions are missing, except row 8, which is a single case row. This case is the only multi-target intervention that is unsuccessful and in which partner or family members were not involved.

**Table 7:** Truth table of multi-target interventions sub-analysis – interventions targeting both women and health providers or systems

Row	Group based delivery	Communication (IEC) materials	Partner or family member involvement	Interaction with health providers	Multi-target intervention	Outcome	Number of cases	inclS*	PRI**	Cases
1	1	1	1	1	1	1	5	1	1	Runmei (2012), Borem (2020), [46] Yu (2017), [45] Xia (2019), [44] Clarke (2020) [48]
2	1	1	1	0	0	1	2	1	1	Sharifirad (2013), [68] Feinberg (2015) [71]
3	1	1	1	1	0	1	2	1	1	Valiani (2014), [69] Rouhe (2013) [37]
4	1	1	0	1	0	1	1	1	1	Bastani (2005) [70]
5	0	1	0	0	0	0	1	0	0	Montgomery (2007) [43]
6	0	1	0	1	0	0	3	0	0	Fraser (1997), Fenwick (2015), [41] Saisto (2001) [42]
7	1	0	0	0	0	0	2	0	0	Masoumi (2016), [39] Navaee (2015) [40]
8	1	1	0	1	1	0	1	0	0	Zhang (2020) [49]

^*^Inclusion score (InclS), also known as consistency, indicates the degree to which the evidence is consistent with the hypothesis that there is sufficient relation between the configuration and the outcome; **Proportional Reduction in Inconsistency (PRI) refers to the extent in which a configuration is sufficient in triggering successful outcome as well as the negation of the outcome

**Fig. 5:**
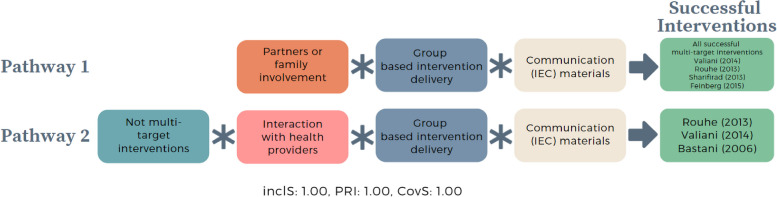
Intermediate pathways from multi-target interventions sub-analysis that trigger successful interventions targeting pregnant women to optimise CS. In QCA, asterisk (*) denotes an ‘AND’ relationship; Inclusion score (InclS), also known as consistency, indicates the degree to which the evidence is consistent with the hypothesis that there is sufficient relation between the configuration and the outcome; Proportional Reduction in Inconsistency (PRI) refers to the extent in which a configuration is sufficient in triggering successful outcome as well as the negation of the outcome; Coverage score (CovS) refers to percentage of cases in which the configuration is valid

The Boolean minimisation identified two intermediate pathways (Fig. 5). The first pathway shows that partner or family involvement, provision of IEC materials, and group-based intervention delivery prompt successful interventions. The first pathway is comprised of all five successful multi-target interventions [44–48] and four of 11 interventions targeting only women [37, 68, 69, 71]. The second pathway shows that when multi-target interventions are absent, but when interaction with health providers is present, alongside provision of IEC materials and group-based intervention delivery, it prompts successful interventions (3/11 interventions targeting women only [37, 69, 70]). The first pathway shows that there are successful configurations with and without multi-target interventions. Therefore, similar to the interventions targeting women, when implementing multi-target interventions, intervention components targeting women are more likely to be successful when partners or family members are involved, interventions are implemented through group-based intervention delivery, IEC materials were provided, and there is an opportunity for women to interact with health providers.

To summarise, there are four essential intervention components which trigger successful educational interventions focusing on pregnant women to reduce CS, this includes 1) group-based intervention delivery, 2) provision of IEC materials on what to expect during labour and birth, 3) partner or family member involvement on the intervention, and 4) opportunity for women to interact with health providers. These conditions do not work in siloed or independently but instead work jointly as parts of configurations to enable successful interventions.

## Discussion

Our extensive QCA identified configurations of essential intervention components which are sufficient to trigger successful interventions to optimised CS. Educational interventions focusing on women were successful by: 1) leveraging social or peer support through group-based intervention delivery, 2) improving women’s knowledge and awareness of what to expect during labour and birth, 3) ensuring women have emotional support through partner or family participation in the intervention, and 4) providing opportunities for women to interact with health providers. We found that the absence of two or more of the above characteristics in an intervention result in unsuccessful interventions. Unlike our logic model, which predicted engagement strategies (i.e. intensity, frequency, technique, recruitment, incentives) to be essential to intervention success, we found that “support” seems to be central in maximising benefits of interventions targeting women.

Group-based intervention delivery is present across all four truth tables and eight pathways leading to successful intervention implementation, suggesting that group-based intervention delivery is an essential component of interventions targeting women. Despite this, we cannot conclude that group-based intervention delivery is a *necessary* condition, as there may be other pathways not captured in this QCA. The importance of group-based intervention delivery may be due to the group setting providing women with a sense of confidence through peer support and engagement. In group-based interventions, women may feel more confident when learning with others and peer support may motivate women. Furthermore, all group-based interventions in our included studies are conducted at health facilities, which may provide women with more confidence that information is aligned with clinical recommendations. Evidence on benefits of group-based interventions involving women who are pregnant has been demonstrated previously [72, 73]. Women reported that group-based interventions reduce their feelings of isolation, provide access to group support, and allow opportunities for them to share their experiences [72, 74–76]. This is aligned with social support theory, in which social support through a group or social environment may provide women with feelings of reassurance, compassion, reduce feelings of uncertainty, increase sense of control, access to new contacts to solve problems, and provision of instrumental support, which eventually influence positive health behaviours [72, 77]. Women may resolve their uncertainties around mode of birth by sharing their concerns with others and learning at the same time how others cope with it. These findings are consistent with the benefits associated with group-based antenatal care, which is recommended by WHO [78, 79].

Kingdon et al. (2018) reported that women and communities liked learning new birth information, as it opens new ways of thinking about vaginal birth and CS, and educates about benefits of different modes of birth, including risks of CS. Our QCA is aligned with this finding where provision of information about birth through education delivery leads to successful interventions but with certain caveats. That is, provision of birth information should be accompanied by IEC materials and through group-based intervention delivery. There is not enough information to distinguish what type of IEC materials lead to successful intervention; however, it is important to note that the format of the IEC materials (such as paper-based or mobile application) may affect success. More work is needed to understand how women and families react to format of IEC materials; for example, will paper-based IEC materials be relegated over more modern methods of reaching women with information through digital applications? The QUALI-DEC (Quality decision-making (QUALI-DEC) by women and healthcare providers for appropriate use of caesarean section) study is currently implementing a decision-analysis tool to help women make an informed decision on preferred mode of birth using both a paper-based and mobile application that may shed some light on this [80].

Previous research has shown that women who participated in interventions aiming to reduce CS desired emotional support (from partners, doulas or health providers) alongside the communication about childbirth [22]. Our QCA is aligned with this finding in which emotional support from partners or family members is highly influential in leading to successful interventions. Partner involvement in maternity care has been extensively studied and has been demonstrated to improve maternal health care utilisation and outcomes [81]. Both women and their partners perceived that partner involvement is crucial as it facilitates men to learn directly from providers, thus promoting shared decision-making among women and partners and enabling partners to reinforce adherence to any beneficial suggestions [82–86]. Partners provide psychosocial support to women, for example through being present during pregnancy and the childbirth process, as well as instrumental support, which includes supporting women financially [82–84]. Despite the benefits of partner involvement, partner's participation in maternity care is still low [82], as reflected in this study where only four out of 11 included interventions on this study involved partner or family member involvement. Reasons for this low participation, which include unequal gender norms and limited health system capability [82, 84–86], should be explored and addressed to ensure the benefits of the interventions.

Furthermore, our QCA demonstrates the importance of interaction with health providers to trigger successful interventions. The interaction of women with providers in CS decision-making, however, is on a “nexus of power, trust, and risk”, where it may be beneficial but can also reinforce the structural oppression of women [13]. A recent study on patient-provider interaction in CS decision-making concluded that the interaction between providers who are risk-averse, and women who are cautious about their pregnancies in the health system results in discouragement of vaginal births [87]. However, this decision could be averted by meaningful communication between women and providers where CS risks and benefits are communicated in an environment where vaginal birth is encouraged [87]. Furthermore, the reasons women desire interaction with providers can come from opposite directions. Some women see providers as the most trusted and knowledgeable source, in which women can trust the judgement and ensure that the information learned is reliable and evidenced-based [22]. On the other hand, some women may have scepticism towards providers where women understand that providers’ preference may negatively influence their preferred mode of birth [22]. Therefore, adequate, two-way interaction is important for women to build a good rapport with providers.

It is also important to note that we have limited evidence (3/17 intervention studies) involving women with previous CS. Vaginal birth after previous CS (VBAC) can be a safe and positive experience for some women, but there are also potential risks depending on their obstetric history [88–90]. Davis (2020) found that women were motivated to have VBAC due to negative experiences of CS, such as the difficult recovery, and that health providers' roles served as pivotal drivers in motivating women towards VBAC [91]. Other than this, VBAC also requires giving birth in a suitably staffed and equipped maternity unit, with staff trained on VBAC, equipment for labour monitoring, and resources for emergency CS if needed [89, 90]. There is comparatively less research conducted on VBAC and trial of labour after CS [88]. Therefore, more work is needed to explore if there are potentially different pathways that lead to successful intervention implementation for women with previous CS. It may be more likely that interventions targeting various stakeholders are more crucial in this group of women. For example, both education for women and partners or families, as well as training to upskill health providers might be needed to support VBAC.

### Strength and limitations

We found many included studies had poor reporting of the interventions, including the general intervention components (e.g. presence of policies that may support interventions) and process evaluation components, which is reflective of the historical approach to reporting trial data. This poor reporting means we could not engage further in the interventions and thus may have missed important conditions that were not reported. However, we have attempted to compensate for limited process evaluation components by identifying all relevant sibling studies that could contribute to a better understanding of context. Furthermore, there are no studies conducted in low-income countries, despite rapidly increasing CS rates in these settings. Lastly, we were not able to conduct more nuanced analyses about CS, such as exploring how CS interventions impacted changes to emergency versus elective CS, VBAC, or instrumental birth, due to an insufficient number of studies and heterogeneity in outcome measurements. Therefore, it is important to note that we are not necessarily measuring the optimal outcome of interest—reducing unnecessary CS. However, it is unlikely that these non-clinical interventions will interfere with a decision of CS based on clinical indications.

Despite these limitations, this is the first study aiming to understand how certain interventions can be successful in targeting women to optimise CS use. We used the QCA approach and new analytical frameworks to re-analyse existing systematic review evidence to generate new knowledge. We ensure robustness through the use of a logic model and worked backwards in understanding what aspects are different in the intervention across different outcomes. The use of QCA and qualitative evidence synthesis ensured that the results are theory-driven, incorporate participants’ perspectives into the analysis, and explored iteratively to find the appropriate configurations, reducing the risk of data fishing. Lastly, this QCA extends the understanding of effectiveness review conducted by Chen et al. (2018) [18] by explaining the potential intervention components which may influence heterogeneity.

### Implications for practice and research

To aid researchers and health providers to reduce CS in their contexts and designing educational interventions targeting women during pregnancy, we have developed a checklist of key components or questions to consider when designing the interventions that may help lead to successful implementation:

1. Is the intervention delivered in a group setting?
2. Are IEC materials on what to expect during labour and birth disseminated to women?
3. Are women’s partners or families involved in the intervention?
4. Do women have opportunities to interact with health providers?

**Fig. 6:**
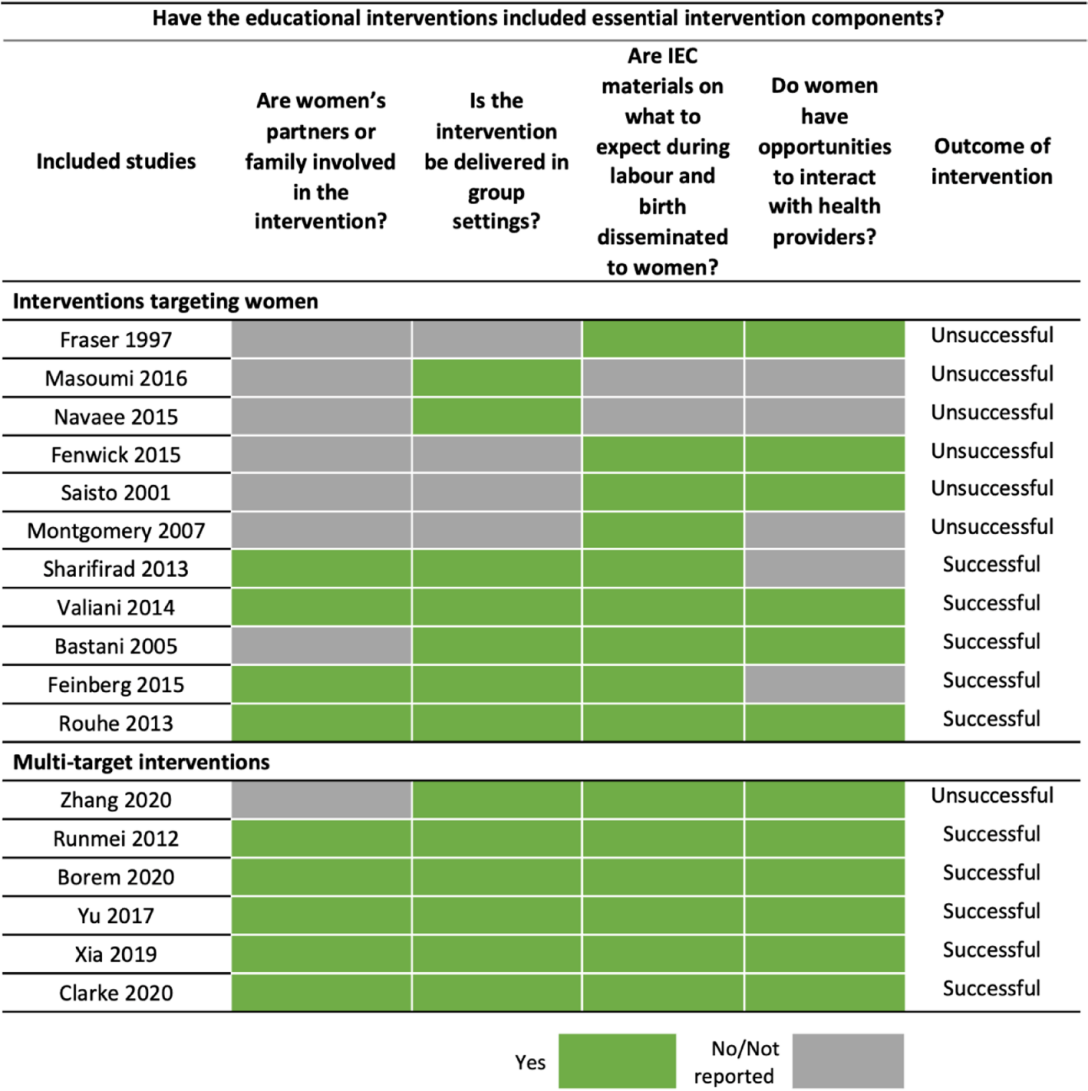
Matrix model assessing the extent to which the included intervention studies have essential intervention components identified in the QCA

We have used this checklist to explore the extent to which the included interventions in our QCA include these components using a matrix model (Fig. 6).

Additionally, future research on interventions to optimise the use of CS should report the intervention components implemented, including process outcomes such as fidelity, attrition, contextual factors (e.g. policies, details of how the intervention is delivered), and stakeholder factors (e.g. women’s perceptions and satisfaction). These factors are important in not just evaluating whether the intervention is successful or not, but also in exploring why similar interventions can work in one but not in another context. There is also a need for more intervention studies implementing VBAC to reduce CS, to understand how involving women with previous CS may result in successful interventions. Furthermore, more studies understanding impact of the interventions targeting women in LMICs are needed.

## Conclusion

This QCA illustrates crucial intervention components and potential pathways that can trigger successful educational interventions to optimise CS, focusing on pregnant women. The following intervention components are found to be sufficient in triggering successful outcomes: 1) group-based delivery, 2) provision of IEC materials, 3) partner or family member involvement, and 4) opportunity for women to interact with health providers. These intervention components do not work in siloed or independently but instead work jointly as parts of configurations to enable successful interventions. Researchers, trialists, hospitals, or other institutions and stakeholders planning interventions focusing on pregnant women can consider including these components to ensure benefits. More studies understanding impact of the interventions targeting women to optimise CS are needed from LMICs. Researchers should clearly describe and report intervention components in trials, and consider how process evaluations can help explain why trials were successful or not. More robust trial reporting and process evaluations can help to better understand mechanisms of action and why interventions may work in one context yet not another.

### Supplementary Information

**Additional file 1.** Logic model in optimizing CS use.

**Additional file 2.** Risk of bias assessments.

**Additional file 3.** Coding framework and calibration rules.

**Additional file 4.** Coding framework as applied to each intervention (data table).

## Data Availability

Additional information files have been provided and more data may be provided upon request to r.zahroh@unimelb.edu.au.

## Abbreviations

CovS: Coverage score
CS: Caesarean section
csQCA: Crisp set qualitative comparative analysis
fsQCA: Fuzzy set qualitative comparative analysis
IEC: Information, education, and communication
InclS: Inclusion score
LMICs: Low- and middle-income countries
PRI: Proportional reduction in inconsistency
QCA: Qualitative comparative analysis
QUALI-DEC: Quality decision-making by women and healthcare providers for appropriate use of caesarean section
VBAC: Vaginal birth after previous caesarean section
WHO: World Health Organization
