# Supplementary figures and images for: Educational interventions targeting pregnant women to optimise the use of caesarean section: What are the essential elements? A qualitative comparative analysis
Source: BMC Public Health. 2023 Sep 23;23:1851. doi: 10.1186/s12889-023-16718-0 (PMC10517530; doi:10.1186/s12889-023-16718-0)

**Additional file 1 – Logic model in optimizing CS use**
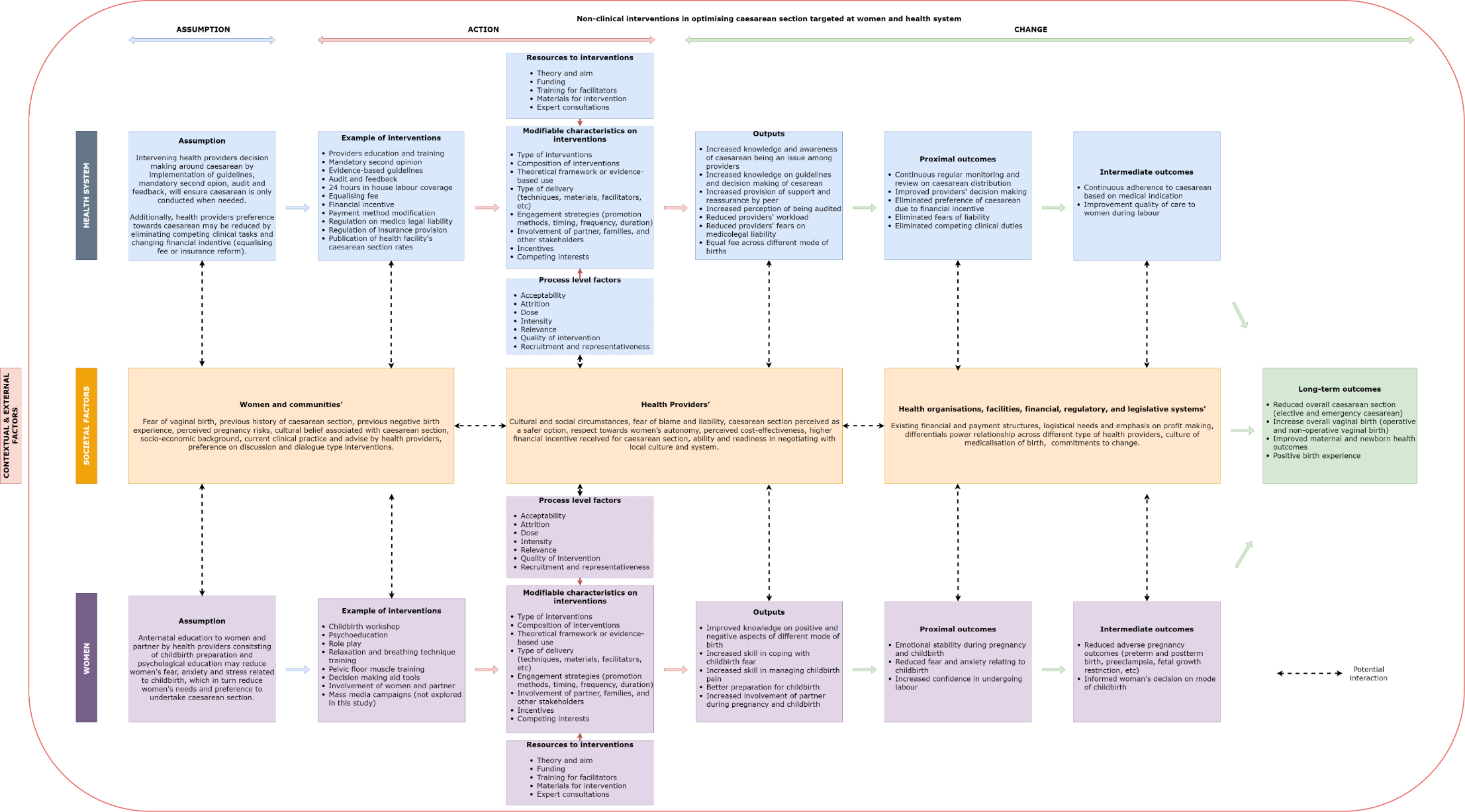

Supplement: Supplementary file 1 — Additional file 1. Logic model in optimizing CS use. [file 12889_2023_16718_MOESM1_ESM.docx]
