# Supplementary material for: Educational interventions targeting pregnant women to optimise the use of caesarean section: What are the essential elements? A qualitative comparative analysis
Source: BMC Public Health. 2023 Sep 23;23:1851. doi: 10.1186/s12889-023-16718-0 (PMC10517530; doi:10.1186/s12889-023-16718-0)
Supplement: Supplementary file 2 — Additional file 2. Risk of bias assessments. [file 12889_2023_16718_MOESM2_ESM.docx]

## **Additional file 2 – Risk of bias assessment – detailed appraisal**

### **Additional file 2.1 – Risk of bias assessment – overview**

| Author | Year | Methods | Risks of Bias | Source | Tools |
| --- | --- | --- | --- | --- | --- |
| Targeting women |  |  |  |  |  |
| Fraser 1997 | 1997 | Randomised controlled trial | Some concerns | Chen et al (2018) | Cochrane EPOC 'Risk of bias' criteria |
| Masoumi 2016 | 2016 | Randomised controlled trial | Some concerns | Chen et al (2018) | Cochrane EPOC 'Risk of bias' criteria |
| Navaee 2015 | 2015 | Blind clinical trial | Some concerns | Chen et al (2018) | Cochrane EPOC 'Risk of bias' criteria |
| Fenwick 2015 | 2015 | Randomised controlled trial | Some concerns | Chen et al (2018) | Cochrane EPOC 'Risk of bias' criteria |
| Saisto 2001 | 2001 | Randomised controlled trial | Some concerns | Chen et al (2018) | Cochrane EPOC 'Risk of bias' criteria |
| Montgomery 2007 | 2007 | Randomised controlled trial | Some concerns | Chen et al (2018) | Cochrane EPOC 'Risk of bias' criteria |
| Sharifirad 2013 | 2013 | Randomised controlled trial | Some concerns | Chen et al (2018) | Cochrane EPOC 'Risk of bias' criteria |
| Valiani 2014 | 2014 | Randomised controlled trial | Some concerns | Chen et al (2018) | Cochrane EPOC 'Risk of bias' criteria |
| Bastani 2005 | 2006 | Randomised controlled trial | Some concerns | Chen et al (2018) | Cochrane EPOC 'Risk of bias' criteria |
| Feinberg 2015 | 2015 | Randomised controlled trial | Some concerns | Chen et al (2018) | Cochrane EPOC 'Risk of bias' criteria |
| Rouhe 2013 | 2013 | Randomised controlled trial | Some concerns | Chen et al (2018) | Cochrane EPOC 'Risk of bias' criteria |
| Multi-target |  |  |  |  |  |
| Xia 2019 | 2019 | Uncontrolled before-after study | Low risks of bias | Current study | Cochrane EPOC 'Risk of bias' criteria |
| Zhang 2020 | 2020 | Randomised controlled trial | Some concerns | Current study | Cochrane Risk of Bias Tool for randomised trials |
| Yu 2017 | 2017 | Pre-post intervention study | Not serious | Opiyo et al (2020) | GRADE |
| Borem 2020 | 2020 | Interrupted time series | Not serious | Opiyo et al (2020) | GRADE |
| Runmei 2012 | 2012 | Controlled before-after  study | Some concerns | Chen et al (2018) | Cochrane EPOC 'Risk of bias' criteria |
| Clarke 2020 | 2020 | Randomised controlled trial | Some concerns | Current study | Cochrane Risk of Bias Tool for randomised trials |

### **Additional file 2.2 – Risk of bias assessment of randomised controlled trial using Cochrane Risk of Bias Tool for randomised trials**

| Components assessed | Zhang (2020) | Clarke (2020) |
| --- | --- | --- |
| Risk of bias arising from the randomization process | Low risk | Some concerns |
| Notes | Allocation random and concealed after assignment. No significant baseline differences | Allocation random and concealed after assignment. Significantly higher number of women in the intervention group with higher number of VB history (before previous births) |
| Risk of bias arising from the timing of identification or recruitment of participants | Low risk | Low risk |
| Notes | Hospitals are recruited before randomisation | Cluster (hospitals) recruited before randomisation but women were recruited after randomisation |
| Risk of bias due to deviations from the intended interventions | Low risk | Some concerns |
| Notes | Participants (women and health providers) are aware of the trials and may be aware of the assigned intervention. It was mentioned that "No masking was applied in this study" | Participants (women and health providers) are aware of the trials and may be aware of the assigned intervention. It was mentioned that "the trial team were blinded to the results of the trial" but not the assigned intervention |
| Risk of bias due to missing outcome data | Low risk | Low risk |
| Notes | Random selection at pre- and post-intervention | No missing outcome data |
| Risk of bias in measurement of the outcome | Some concerns | Some concerns |
| Notes | Unclear if the outcomes assessor is blinded or not | Unclear if the outcomes assessor is blinded or not |
| Risk of bias in selection of the reported result | Low risk | Low risk |
| Notes | Seems to be intention to treat analysis. Results are not assessed based on multiple measurements or multiple analysis | Seems to be intention to treat analysis. Results are not assessed based on multiple measurements or multiple analysis |
| Final Assessment | Some concerns | Some concerns |

### **Additional file 2.3 – Risk of bias assessment of uncontrolled before and after study using Cochrane EPOC 'Risk of bias' criteria**

| Components assessed | Xia (2019) |
| --- | --- |
| Intervention independent of other changes | Low risk |
| Notes | Covariance assessed |
| Shape of the intervention effect pre-specified | Low risk |
| Notes | Point of intervention and analysis seems relevant |
| Intervention unlikely to affect data collection | Low risk |
| Notes | Sources and methods of data collection were the same before and after the intervention: using medical records |
| Knowledge of the allocated interventions adequately prevented during the study | Low risk |
| Notes | Outcomes are objectively assessed |
| Incomplete outcome data (attrition bias) | Low risk |
| Notes | 1000 out of almost 2,000,000 data |
| Selective outcome reporting (reporting bias) | Low risk |
| Notes | All relevant outcomes in the methods section are reported in the results section |
| Other risks of bias | N/A |
| Notes | N/A |
| Final Assessment | Low risk of bias |
