# Supplementary material for: Educational interventions targeting pregnant women to optimise the use of caesarean section: What are the essential elements? A qualitative comparative analysis
Source: BMC Public Health. 2023 Sep 23;23:1851. doi: 10.1186/s12889-023-16718-0 (PMC10517530; doi:10.1186/s12889-023-16718-0)
Supplement: Supplementary file 3 — Additional file 3. Coding framework and calibration rules. [file 12889_2023_16718_MOESM3_ESM.docx]

## **Additional file 3 – Coding and calibration rules**

| **Condition** | **Field** | **Instructions for extractors** | **Calibration type** | **Coding values and calibration rules** |
| --- | --- | --- | --- | --- |
| **Domain 1** | **Setting and participants** | |  |  |
| **1.1** | Number of participants | Recorded total number of participants involved in intervention (record only the number of people that received the intervention) | Transformational assignment (Ragin’s direct method) | 100 or more participants = 0  75 participants = 0.5 50 or fewer = 1  Other values span between 0 to 1 |
| **1.2** | Composition of participants | What were the composition of participants? Is it only women, or women and partners? | Direct assignment | Partner or family members involvement: yes = 1, no = 0  Women with fear/anxiety of birth: yes = 1, no = 0  General pregnant women: yes = 1, no = 0  Women with previous CS: yes = 1, no = 0  When we code this, we considered partner or family members involvement with “involvement of other stakeholders” condition 4.2 |
| **1.3** | Intervention location | Did the interventions delivered at health facilities, home, or communities? | Direct assignment | Health facility = yes: 1, no: 0  Home or communities = yes: 1, no: 0 |
| **1.4** | Number of health facility | Recorded number of health facilities implementing the intervention | Transformational assignment (Ragin’s direct method) | 10 or more health facilities = 0  7 health facilities = 0.5  5 or fewer = 1  Other values span between 0 to 1 |
| **1.5** | Number of health facility | Recorded number of health facilities implementing the intervention | Transformational assignment (Ragin’s direct method) | 10 or more health facilities = 0  7 health facilities = 0.5 5 or fewer = 1  Other values span between 0 to 1 |
| **1.6** | Baseline CS rates | Recorded baseline CS rates at the health facility | Direct assignment | Take into account if they considered baseline CS rates at facility/area/country for implementing the intervention  High baseline CS rates (>15%) at study facility = 1  High baseline CS rates (>15%) at area/country= 0.66  Not reported = 0  Not considering baseline rates but mention high preference of CS/increasing CS = 0.33  Not considering CS at all OR low based CS rates<10% CS rates = 0 |
| **Domain 2** | **Intervention design** |  |  |  |
| **2.1** | Component of interventions | Extract the type of interventions included: i.e antenatal education, psychosocial education, audit and feedback implementation | Direct assignment to all listed interventions | Any type of education? (including ante, psycho and decision aid): yes = 1, no = 0  Antenatal education (including childbirth classes, couple education): yes = 1, no = 0 Pyscho education/therapy (including cognitive therapy, relaxation/breathing technique): yes = 1, no = 0 Decision aids (information leaflet, website): yes = 1, no = 0  Acknowledgement of women's previous birth experience: yes = 1, no = 0 |
| **2.2** | Utilisation of theoretical framework or evidence-based intervention | Did the study name a theoretical framework that underpins the intervention design or delivery style? | Direct assignment | Theory driven = 1  Citing previous study only = 0.25 Missing/Not using any/ambiguous = 0 |
| **2.3** | Type of intervention facilitators or instructors | Who were the instructors or facilitators of the intervention? i.e. peers, obstetrician, midwives, doula, other trained staff | Direct assignment to all listed facilitators | Maternity health providers (nurse, midwife, obstetrician): yes = 1; not mentioned as an instructor/facilitator = 0  Any interaction with health providers? Both in individual and group settings: yes = 1, no = 0  Opportunity of having interaction with health providers in group settings: yes = 1, no = 0, not reported = 0 |
| **2.4** | Personal or group delivery | Did the intervention delivered in a group or personal based delivery through workshop or class? | Direct assignment | Group delivery: yes = 1, no = 0  Personalised/individualised: yes = 1, no = 0 |
| **2.5** | Type of materials used | Did the intervention used specific type of materials? i.e. video, computer-based tool, pamphlet, handbook, guidelines | Direct assignment to all listed materials | IEC materials (written, audio, video, decision aids): yes = 1, no/not reported = 0  IEC materials can be taken home by women: yes = 1, no/not reported = 0 |
| **2.7** | Resources required | What were the cost required to implement and deliver the intervention? | Direct assignment | Cost less than usual care = 1  Cost more the same as usual care = 0.66  Cost more than usual care = 0.33  Not reported= 0 |
| **2.8** | Information about control condition | Described whether the authors provided a control for the main intervention (intended to capture complexity of running an intervention and a control) - are they equivalent or not? | Transformational assignment, 1 equal to equivalent control | No control described = 0 Ambiguous = 0.33 Yes, but not an equivalent = 0.66 Yes, an equivalent control = 1 |
| **Domain 3** | **Program content** |  |  |  |
| **3.1** | Content delivered to mother and partners | What type of content themes or curriculum delivered to mother and partners? i.e. relaxation technique, information on vaginal birth vs caesarean section risks | Direct assignment to all listed content | Topic focus - general childbirth: yes = 1, no = 0  Topic focus - fear and anxiety: yes = 1, no = 0  Information about mode of birth including delivery process: yes = 1, no = 0 Pain and pain relief: yes = 1, no = 0 Mental health and coping strategies: yes = 1, no = 0 Partner's role and communication: yes = 1, no = 0 |
| **3.4** | Educational or training technique | What were the education or training technique used? i.e. role-play, lecture-based, study cases | Direct assignment to all listed content | Practice based (practical session, behavioural rehearsal, role play): yes = 1, no = 0, not reported = 0 Lecture/didactic based: yes = 1, no = 0, not reported = 0 |
| **Domain 4** | **Engagement** |  |  |  |
| **4.1** | Methods of recruitment | How were participants recruited to the study? i.e. flyers, snowballing, hospital-based recruitment | Direct assignment to all listed content | Recruited at health facility (enrollment at certain programs, health providers, registry, facility/provider office, phone call): yes = 1, no = 0, not reported = 0  Advertisements (media, flyers, word of mouth): yes = 1, no = 0, not reported = 0) |
| **4.2** | Involvement with partners, families, other stakeholders | Other than participants, were there any other stakeholders included in the intervention? | Direct assignment | Yes = 1 No = 0  This code was merged with composition of participants condition 1.2 on the first page |
| **4.3** | Timing of engagement | In which pregnancy stage the intervention was delivered? i.e. first trimester, last trimester, before pregnancy, at hospital admission for birth, after birth, prior to pregnancy | Direct assignment to all listed content | Antenatal: yes = 1, no = 0  Intrapartum: yes = 1, no = 0, no int delivered during intrapartum  Postnatal: yes = 1, no = 0 |
| **4.4** | Frequency of engagement | How often did the intervention delivered (or frequency of engagement/encounter between participants and intervention)? i.e. once in a month, every 3 months | Direct assignment | No engagement= 0  1-time contact = 0.33  Ambiguous/not reported = 0  2 times contacts = 0.66  3 or more times/contacts = 1 |
| **4.5** | Required time engaged in the interventions | Record any statement or recommendation from the authors as to how long the interventions had or should occurred (i.e during the course of the pregnancy, hours required) | Direct assignment | No engagement/not reported = 0 5 hours or fewer = 0.33  5 - 10 hours or more = 0.66 10 hours or more = 1 |
| **4.6** | Existing competing interest | Was there any competing interest stated? | Direct assignment | No = 1  Yes/ Not reported = 0 |
| **Domain 5** | **Health system** |  |  |  |
| **5.1** | Presence of internal policies | Were there any existing policies in place to support the intervention (both at facility, regional, or country level)? | Direct assignment | No = 0  Not reported = 0 Yes = 1 |
| **5.2** | Presence of any other support and resources | Were there any existing support from health system which directly or indirectly support the intervention? | Direct assignment | No = 0  Not reported = 0 Yes = 1 |
| **5.3** | Perceptions of stakeholders related to the interventions or the caesarean section itself | What were the perceptions of both participants and other relevant stakeholders in regard to caesarean section? | Direct assignment | Missing/not reported = 0 Negative = 0.33 Medium/neutral = 0.5  More positive, despite few dissatisfaction = 0.66 Positive = 1 |
| **Domain 6** | **Process outcomes** |  |  |  |
| **6.1** | Intervention fidelity | Extract level of fidelity (%) or extract qualitative statement on intervention fidelity. Intervention fidelity refers to the degree to which the prescribed components of the intervention, as described in the study protocol, have been delivered | Direct assignment | There is evidence on high fidelity = 1 Med fidelity 0.66 Low fidelity = 0.33 Not reported = 0 |
| **6.2** | Participants satisfaction | Extract level of satisfaction (%) or extract qualitative statement on participants' satisfaction with the intervention experience | Direct assignment | Direct assignment - qualitative: where there is a positive statement = 0.66; negative statement = 0.33; where not mentioned = 0  Direct assignment - quantitative: Interventions with 25% or fewer participants satisfied = 0; with 50% of participants satisfied = 0.5; not mentioned = 0; interventions with >75% participants satisfied = 1 |
| **6.3** | Participants attrition | Extract participants' level of completion (%) or record qualitative statement on participants' completion rate | Direct assignment | Direct assignment - qualitative: where there is a statement indicating high level of completion, assign value of 0.66; where a statement indicating problematic completion, assign value of 0.33. Where data are missing, assign value of 0  Direct assignment - quantitative: Interventions with 66% or fewer participants completing the intervention = 0; interventions with 75% of participants completing the intervention = 0.5; interventions with 83% or more participants completing the intervention = 1. Missing data coded as 0 |
| **6.4** | Participants dosage level | Did the participant receive the intended dosage of the intervention? "intended dosage is the amount of the components of the intervention delivered by implementers and the extent to which participants received and used materials or other resources". Extract level of dosage (%) or record qualitative statement | Direct assignment | Direct assignment - qualitative: where there is a qualitative statement indicating high level of dosage, assign value of 0.66; where a qualitative statement indicating problematic dosage, assign value of 0.33. Where data are missing, assign value of 0  Direct assignment - quantitative: Interventions with 66% or fewer participants receiving the full dosage = 0; interventions with 75% of participants receiving the full dosage = 0.5; interventions with 83% or more of participants receiving the full dosage = 1. Missing data coded as 0 |
| **6.5** | Participant adherence | Did participants adhere to the intervention instructions, e.g. women and partners attending classes; health providers conducted mandatory second opinion. Extract level of adherence (%) or qualitative statement |  | Direct assignment - qualitative: where there is a statement indicating high level of adherence, assign value of 0.66; where a qualitative statement indicating problematic adherence, assign value of 0.33. Where data are missing, assign value of 0  Direct assignment - quantitative: Interventions with 66% or fewer participants adherent = 0; interventions with 75% of participants adherent = 0.5; interventions with 83% or more participants adherent = 1. Missing data coded as 0 |
| **6.6** | Certainty of evidence | Certainty or confidence in which we can place into finding | Direct assignment | Moderate = 1  Low or very low = 0 |
| **6.7** | Collateral effect other than to participants | Is there any stated collateral effects on others (other than participants)? i.e. if women obtain 24/7 epidural but no additional human resources are provided to the unit, doctors may need to do more shifts or work longer hours to their in satisfaction. | Not coded | Not coded only for consideration whether the intervention is successful or not |
| **6.8** | Any other factors affecting the success/unsuccessful of the implementation? | Any other factors affecting the success/unsuccessful of the implementation? | Not coded | Not coded only for consideration whether the intervention is successful or not |
| **Domain 7** | **Type of interventions** |  |  |  |
| **7.1** | Type of interventions | What is the type of interventions? Is it interventions targeting women or multi-target interventions? | Direct assignment | Interventions targeting women: yes = 1, no = 0  Multi-target interventions: yes = 1, no = 0 |
