# Supplementary material for: Educational interventions targeting pregnant women to optimise the use of caesarean section: What are the essential elements? A qualitative comparative analysis
Source: BMC Public Health. 2023 Sep 23;23:1851. doi: 10.1186/s12889-023-16718-0 (PMC10517530; doi:10.1186/s12889-023-16718-0)
Supplement: Supplementary file 4 — Additional file 4. Coding framework as applied to each intervention (data table). [file 12889_2023_16718_MOESM4_ESM.docx]

## **Additional file 4 – Coding framework as applied to each intervention (data table)**

| Domain | Conditions | Fraser 1997 | Masoumi 2016 | Navaee 2015 | Fenwick 2015 | Saisto 2001 | Montgomery 2007 | Sharifirad 2013 | Valiani 2014 | Bastani 2005 | Feinberg 2015 | Rouhe 2013 | Runmei 2012 | Borem 2020 | Yu 2017 | Xia 2019 | Zhang 2020 | Clarke 2020 |
| --- | --- | --- | --- | --- | --- | --- | --- | --- | --- | --- | --- | --- | --- | --- | --- | --- | --- | --- |
| Domain 1 - settings and participants | Number of participants | 0 | 0 | 0.991085 | 0 | 0.235452 | 0 | 0.974695 | 0.854042 | 0.01595 | 0.16126 | 0.001365 | 0 | 0 | 0 | 0 | 1 | 0 |
| Domain 1 - settings and participants | Partner/families involvement | 0 | 0 | 0 | 0 | 0 | 0 | 1 | 1 | 0 | 1 | 1 | 1 | 1 | 1 | 1 | 0 | 1 |
| Domain 1 - settings and participants | Women with fear | 0 | 0 | 1 | 1 | 1 | 0 | 0 | 0 | 1 | 0 | 1 | 0 | 0 | 0 | 0 | 0 | 0 |
| Domain 1 - settings and participants | Women with low risks pregnancy | 0 | 1 | 0 | 0 | 0 | 0 | 1 | 1 | 0 | 1 | 0 | 1 | 1 | 1 | 1 | 1 | 0 |
| Domain 1 - settings and participants | Women with previous CS | 1 | 0 | 0 | 0 | 0 | 1 | 0 | 0 | 0 | 0 | 0 | 0 | 0 | 0 | 0 | 0 | 1 |
| Domain 1 - settings and participants | Intervention location - health facility | 1 | 1 | 1 | 0 | 1 | 0 | 1 | 1 | 1 | 1 | 1 | 1 | 1 | 1 | 1 | 1 | 1 |
| Domain 1 - settings and participants Domain 1 - settings and participants | Intervention location - Home or community | 0 | 0 | 0 | 1 | 0 | 1 | 0 | 0 | 0 | 0 | 0 | 0 | 0 | 0 | 1 | 0 | 0 |
| Domain 1 - settings and participants | Number of health facility | 0.007337 | 0.999854 | 0.95 | 1 | 0.999854 | 1 | 0.999854 | 0 | 0.997238 | 0 | 0.999854 | 1 | 0 | 1 | 0 | 0 | 0 |
| Domain 1 - settings and participants | Baseline CS rates | 0.33 | 0.66 | 0.66 | 0.33 | 0.33 | 1 | 0.66 | 0.66 | 0.33 | 0 | 0.66 | 1 | 1 | 1 | 1 | 1 | 1 |
| Domain 2 - intervention designs | Any type of education? (including ante, psycho and decision aid) | 1 | 1 | 1 | 1 | 1 | 0 | 1 | 1 | 1 | 1 | 1 | 1 | 1 | 1 | 1 | 1 | 1 |
| Domain 2 - intervention designs | Antenatal education | 1 | 0 | 1 | 0 | 0 | 0 | 1 | 1 | 0 | 0 | 0 | 1 | 1 | 1 | 1 | 1 | 1 |
| Domain 2 - intervention designs | Psychoeducation/therapy (including breathing and relaxation technique) | 0 | 1 | 0 | 1 | 1 | 0 | 0 | 0 | 1 | 1 | 1 | 0 | 0 | 0 | 0 | 0 | 0 |
| Domain 2 - intervention designs | Decision aids | 0 | 0 | 0 | 1 | 0 | 1 | 0 | 0 | 0 | 0 | 0 | 0 | 0 | 0 | 0 | 0 | 0 |
| Domain 2 - intervention designs | Acknowledgement of women's previous birth experience | 1 | 0 | 0 | 0 | 1 | 0 | 0 | 0 | 0 | 0 | 0 | 0 | 1 | 0 | 0 | 0 | 0 |
| Domain 2 - intervention designs | Utilisation of theoretical framework or evidence-based intervention | 1 | 0.25 | 0.25 | 1 | 1 | 0.25 | 0.25 | 0.25 | 1 | 1 | 1 | 0.25 | 1 | 0.25 | 0.25 | 0.25 | 0.25 |
| Domain 2 - intervention designs | Any interaction with health providers? Both in individual and group settings | 1 | 0 | 0 | 1 | 1 | 0 | 0 | 1 | 1 | 0 | 1 | 1 | 1 | 1 | 1 | 1 | 1 |
| Domain 2 - intervention designs | Opportunity of having interaction with health providers in group settings | 0 | 0 | 1 | 0 | 0 | 0 | 0 | 0 | 1 | 0 | 1 | 0 | 1 | 1 | 1 | 1 | 1 |
| Domain 2 - intervention designs | Facilitator- Maternity health providers (nurse, midwife, obstetrician) | 1 | 0 | 1 | 1 | 1 | 0 | 0 | 0 | 1 | 0 | 0 | 0 | 1 | 1 | 1 | 1 | 1 |
| Domain 2 - intervention designs | Group delivery | 0 | 1 | 1 | 0 | 0 | 0 | 1 | 1 | 1 | 1 | 1 | 1 | 1 | 1 | 1 | 1 | 1 |
| Domain 2 - intervention designs | Personalised/individualised delivery | 1 | 0 | 0 | 1 | 1 | 0 | 0 | 0 | 0 | 0 | 0 | 0 | 1 | 0 | 0 | 1 | 0 |
| Domain 2 - intervention designs | IEC materials (including written, audio, video, with decision aid) | 1 | 0 | 0 | 1 | 1 | 1 | 1 | 1 | 1 | 1 | 1 | 1 | 1 | 1 | 1 | 1 | 1 |
| Domain 2 - intervention designs | IEC materials can be taken home | 0.75 | 0 | 0 | 0.75 | 0.75 | 1 | 0.75 | 0.75 | 0.75 | 0 | 0 | 0.75 | 0 | 0.75 | 1 | 1 | 1 |
| Domain 2 - intervention designs | Resources required | 0 | 0 | 0 | 0.33 | 0 | 0 | 0 | 0 | 0 | 0 | 0.33 | 0 | 0 | 0 | 0 | 0 | 0 |
| Domain 2 - intervention designs | Information about control condition | 0.66 | 1 | 0.33 | 1 | 1 | 1 | 1 | 1 | 1 | 1 | 1 | 0.33 | 1 | 0.33 | 0.33 | 1 | 1 |
| Domain 3 - program content | Topic focus - general childbirth | 1 | 1 | 1 | 1 | 1 | 1 | 1 | 1 | 1 | 1 | 1 | 1 | 1 | 1 | 1 | 1 | 1 |
| Domain 3 - program content | Topic focus - fear and anxiety | 0 | 1 | 0 | 1 | 0 | 0 | 0 | 1 | 1 | 1 | 1 | 0 | 0 | 0 | 0 | 0 | 0 |
| Domain 3 - program content | Content delivered to mother and partners - Information about mode of birth including delivery process | 1 | 1 | 1 | 0 | 1 | 1 | 1 | 1 | 0 | 0 | 0 | 1 | 1 | 1 | 1 | 1 | 1 |
| Domain 3 - program content | Content delivered to mother and partners - mental health and coping strategies | 0 | 1 | 0 | 1 | 0 | 0 | 0 | 1 | 1 | 0 | 0 | 0 | 0 | 0 | 0 | 0 | 0 |
| Domain 3 - program content | Content delivered to mother and partners - Pain and pain relief | 1 | 1 | 0 | 0 | 1 | 0 | 0 | 1 | 1 | 1 | 1 | 0 | 0 | 0 | 0 | 0 | 0 |
| Domain 3 - program content | Content delivered to mother and partners - Partner's roles and communication | 0 | 1 | 0 | 0 | 0 | 0 | 0 | 1 | 0 | 1 | 0 | 0 | 0 | 0 | 0 | 0 | 0 |
| Domain 3 - program content | Educational or training technique - Practice based (practical session, behavioral rehearsal, role play) | 0 | 1 | 1 | 0 | 0 | 0 | 0 | 1 | 1 | 1 | 0 | 0 | 1 | 1 | 0 | 0 | 0 |
| Domain 3 - program content | Educational or training technique - Lecture/didactic based | 0 | 1 | 0 | 0 | 0 | 0 | 1 | 1 | 0 | 1 | 0 | 1 | 1 | 1 | 1 | 1 | 1 |
| Domain 4 - engagement | Methods of recruitment - Recruited at health facility (enrollment at certain programs, health providers, registry, facility/provider office, phone call) | 1 | 0 | 0 | 1 | 1 | 1 | 1 | 0 | 1 | 1 | 1 | 1 | 1 | 1 | 1 | 1 | 1 |
| Domain 4 - engagement | Methods of recruitment - Advertisements (media, flyers, word of mouth) | 0 | 0 | 0 | 0 | 0 | 0 | 0 | 0 | 0 | 1 | 0 | 0 | 0 | 0 | 0 | 0 | 0 |
| Domain 4 - engagement | Timing of engagement - Ante | 1 | 1 | 1 | 1 | 1 | 1 | 1 | 1 | 1 | 1 | 1 | 1 | 1 | 1 | 1 | 1 | 1 |
| Domain 4 - engagement | Timing of engagement - Post | 0 | 0 | 0 | 0 | 0 | 0 | 0 | 0 | 0 | 1 | 1 | 1 | 1 | 1 | 1 | 1 | 0 |
| Domain 4 - engagement | Frequency of engagement | 0.66 | 1 | 0.33 | 0.66 | 1 | 0 | 0.33 | 1 | 1 | 1 | 1 | 0 | 1 | 0.66 | 0 | 0 | 0.66 |
| Domain 4 - engagement | Required time engaged in the interventions | 0 | 1 | 0.33 | 0.33 | 0.66 | 0 | 0.33 | 1 | 1 | 0 | 1 | 1 | 1 | 0 | 1 | 1 | 0.66 |
| Domain 4 - engagement | Existing competing interest | 0 | 1 | 0 | 1 | 0 | 1 | 1 | 1 | 0 | 0 | 1 | 0 | 0 | 0 | 0 | 0 | 0 |
| Domain 4 - engagement | Incentives of any type | 0 | 0 | 0 | 0 | 0 | 0 | 0 | 0 | 0 | 1 | 0 | 0 | 0 | 0 | 0 | 1 | 1 |
| Domain 5 - health system factors | Presence of internal policies | 0 | 0 | 0 | 0 | 0 | 0 | 0 | 0 | 0 | 0 | 0 | 0 | 0 | 1 | 1 | 0 | 1 |
| Domain 5 - health system factors | Presence of any other support and resources | 0 | 0 | 0 | 0 | 0 | 0 | 0 | 0 | 0 | 0 | 0 | 0 | 1 | 0 | 0 | 0 | 1 |
| Domain 5 - health system factors | Perceptions of stakeholders related to the interventions or the caesarean section itself | 0 | 0 | 0 | 0 | 0 | 0.33 | 0 | 0 | 0 | 0 | 0 | 0 | 0 | 0 | 0 | 0 | 0.5 |
| Domain 6 - Participants satisfaction and fidelity | Intervention fidelity | 0 | 0 | 0 | 0 | 0 | 1 | 0 | 0 | 0 | 1 | 0 | 0 | 0 | 0 | 0 | 0 | 1 |
| Domain 6 - Participants satisfaction and fidelity | Participants satisfaction | 0.5 | 0 | 0 | 0 | 0 | 1 | 0 | 0 | 0 | 0 | 0 | 0 | 0 | 0 | 0 | 0 | 1 |
| Domain 6 - Participants satisfaction and fidelity | Participants attrition | 0 | 1 | 0 | 0 | 0 | 1 | 0 | 0 | 1 | 1 | 0 | 0 | 0.66 | 0 | 0 | 0 | 1 |
| Domain 6 - Participants satisfaction and fidelity | Participants dosage level | 0 | 0 | 0 | 0 | 0 | 0 | 0 | 0 | 0 | 1 | 0 | 0 | 0 | 0 | 0 | 0 | 0 |
| Domain 6 - Participants satisfaction and fidelity | Participant adherence | 0 | 1 | 0 | 1 | 0 | 0 | 0 | 0 | 0 | 0 | 0 | 0 | 0 | 0 | 0 | 0 | 0 |
| Domain 6 - Participants satisfaction and fidelity | Certainty of evidence | 1 | 1 | 0 | 0 | 0 | 1 | 0 | 0 | 0 | 0 | 0 | 0 | 1 | 1 | 1 | 0 | 0 |
| Domain 7 - Type of interventions | Multi-target interventions | 0 | 0 | 0 | 0 | 0 | 0 | 0 | 0 | 0 | 0 | 0 | 1 | 1 | 1 | 1 | 1 | 1 |
| Domain 7 - Type of interventions | Interventions targeting women | 1 | 1 | 1 | 1 | 1 | 1 | 1 | 1 | 1 | 1 | 1 | 0 | 0 | 0 | 0 | 0 | 0 |
| Outcome | CS outcome | 0 | 0 | 0 | 0 | 0 | 0 | 1 | 1 | 1 | 1 | 1 | 1 | 1 | 1 | 1 | 0 | 1 |

### **Additional file 4.1 – Coding framework on the final models presented**

| Author | CS outcome | Communication (IEC) materials | Partner or family member involvement | Group based intervention | Antenatal education | Psychoeducation | Interaction with health providers | Multi-target intervention |
| --- | --- | --- | --- | --- | --- | --- | --- | --- |
| Fraser 1997 | 0 | 1 | 0 | 0 | 1 | 0 | 1 | 0 |
| Masoumi 2016 | 0 | 0 | 0 | 1 | 0 | 1 | 0 | 0 |
| Navaee 2015 | 0 | 0 | 0 | 1 | 1 | 0 | 0 | 0 |
| Fenwick 2015 | 0 | 1 | 0 | 0 | 0 | 1 | 1 | 0 |
| Saisto 2001 | 0 | 1 | 0 | 0 | 0 | 1 | 1 | 0 |
| Montgomery 2007 | 0 | 1 | 0 | 0 | 0 | 0 | 0 | 0 |
| Sharifirad 2013 | 1 | 1 | 1 | 1 | 1 | 0 | 0 | 0 |
| Valiani 2014 | 1 | 1 | 1 | 1 | 1 | 0 | 1 | 0 |
| Bastani 2005 | 1 | 1 | 0 | 1 | 0 | 1 | 1 | 0 |
| Feinberg 2015 | 1 | 1 | 1 | 1 | 0 | 1 | 0 | 0 |
| Rouhe 2013 | 1 | 1 | 1 | 1 | 0 | 1 | 1 | 0 |
| Zhang 2020 | 0 | 1 | 0 | 1 | 1 | 0 | 1 | 1 |
| Runmei 2012 | 1 | 1 | 1 | 1 | 1 | 0 | 1 | 1 |
| Borem 2020 | 1 | 1 | 1 | 1 | 1 | 0 | 1 | 1 |
| Yu 2017 | 1 | 1 | 1 | 1 | 1 | 0 | 1 | 1 |
| Xia 2019 | 1 | 1 | 1 | 1 | 1 | 0 | 1 | 1 |
| Clarke 2020 | 1 | 1 | 1 | 1 | 1 | 0 | 1 | 1 |
